# Real‐world clinical, biomarker, and imaging outcomes of lecanemab in Alzheimer's disease: a multicenter longitudinal cohort study in Jiangsu, China

**DOI:** 10.1002/alz.71936

**Published:** 2026-10-26

**Authors:** Limoran Tang, Xicheng Chen, Li Zhang, Lin Fan, Nihong Chen, Chaosheng Li, Zenglin Cai, Guofang Chen, Haifeng Chen, Min Ye, Huifen Zheng, Qi Li, Zhihong Ke, Zheqi Hu, Maohong Cao, Dan Yang, Shuai Xu, Chunming Xie, Xiaobo Li, Xiaotian Xu, Ming Yu, Xuegan Lian, Hua Hu, Hongfei Li, Xiuying Cai, Hong Chen, Wu Xu, Guiyun Cui, Yang Zhao, Jingde Dong, Lili Zhang, Xiaolei Zhu, Hui Zhao, Yun Xu

**Affiliations:** ^1^ Department of Neurology Nanjing Drum Tower Hospital, Affiliated Hospital of Medical School, Nanjing University Nanjing China; ^2^ Jiangsu Stroke Association Nanjing Jiangsu China; ^3^ Department of Biostatistics School of Public Health Nanjing Medical University Nanjing Jiangsu China; ^4^ Department of Geriatrics Nanjing Brain Hospital affiliated to Nanjing Medical University Nanjing China; ^5^ Department of Neurology Taizhou School of Clinical Medicine The Affiliated Taizhou People's Hospital of Nanjing Medical University Nanjing Medical University Taizhou Jiangsu China; ^6^ Department of Neurology Nanjing First Hospital Nanjing Medical University Nanjing China; ^7^ Department of Neurology Affiliated Hospital of Jiangnan University Wuxi Jiangsu China; ^8^ Department of Neurology The Affiliated Suzhou Hospital of Nanjing University Medical School Suzhou China; ^9^ Department of Neurology Xuzhou Central Hospital Jiangsu China; ^10^ Department of Neurology BENQ Medical Center Nanjing China; ^11^ Department of Neurology Geriatric Hospital of Nanjing Medical University Nanjing China; ^12^ Department of Neurology Affiliated Hospital of Nantong University Nantong China; ^13^ Department of Neurology Affiliated ZhongDa Hospital, School of Medicine, Southeast University Nanjing Jiangsu China; ^14^ Department of Neurology Affiliated Subei People's Hospital Yangzhou University Yangzhou China; ^15^ Department of Neurology The Affiliated Hospital of Yangzhou University Yangzhou China; ^16^ Department of Neurology The Affiliated Hospital of Jiangsu University Zhenjiang Jiangsu China; ^17^ Department of Neurology The Third Affiliated Hospital of Soochow University Changzhou Jiangsu China; ^18^ Department of Neurology The Second Affiliated Hospital of Soochow University Suzhou China; ^19^ Department of Neurology The Fourth Affiliated Hospital of Nanjing Medical University Nanjing Jiangsu China; ^20^ Department of Neurology The First Affiliated Hospital of Soochow University Suzhou Medical College of Soochow University Suzhou China; ^21^ Department of Neurology Nanjing First Hospital Nanjing Medical University Nanjing Jiangsu China; ^22^ Department of Neurology The Taixing People's Hospital Taixing China; ^23^ Department of Neurology Affiliated Hospital of Xuzhou Medical University Xuzhou Jiangsu China; ^24^ State Key Laboratory of Pharmaceutical Biotechnology and Institute of Translational Medicine for Brain Critical Diseases Nanjing University Nanjing China; ^25^ Center for Central Nervous Diseases and Neuroscience in Nanjing Drum Tower Hospital Nanjing China; ^26^ Nanjing Key Laboratory for Cardiovascular Information and Health Engineering Medicine Nanjing China; ^27^ Nanjing Neurology Clinical Medical Centre, and Centre for Central Nervous Disease and Neuroscience Nanjing China

**Keywords:** amyloid‐related imaging abnormalities (ARIA), cognitive function, early Alzheimer's disease, lecanemab, plasma biomarkers, real‐world study

## Abstract

**INTRODUCTION:**

Real‐world data on lecanemab's efficacy and the longitudinal dynamics of plasma biomarkers are scarce in Chinese patients with Alzheimer's disease (AD).

**METHODS:**

In this multicenter cohort study, 181 AD patients received biweekly lecanemab. Disease‐stage‐specific Clinical Dementia Rating–Sum of Boxes meaningful within‐patient change defined response at Months 3, 6, and 12. We used penalized models, receiver operating characteristic analysis, attrition comparisons, and exploratory Month 3 plasma biomarker analysis.

**RESULTS:**

Cognitive response emerged at Month 6 and intensified by Month 12, largely restricted to mild cognitive impairment and mild dementia due to AD. Repeated five‐fold cross‐validated areas under the curve across the follow‐up timepoints were 0.560, 0.747, and 0.773, with apolipoprotein E ε4 positivity and more severe cognitive impairment linked to lower response odds. Plasma phosphorylated tau 217 (p‐tau217) and amyloid beta (Aβ) 42/40 altered as early as Month 3. Month 3 Aβ42/40 showed modest exploratory discrimination. The incidence of amyloid‐related imaging abnormalities was 7.2%, all asymptomatic.

**DISCUSSION:**

Lecanemab is effective and safe in AD over 6 to 12 months. Early biomarker changes (p‐tau217, Aβ42/40) precede clinical improvement, serving as scalable response indicators.

## Highlights

- Disease‐stage‐specific CDR‐SB response increased from 6.6% at Month 3 to 44.1% at Month 12.
- Month 12 response occurred in 50.0% of MCI and 66.7% of mild dementia participants, but not in the moderate to severe subgroup.
- Repeated five‐fold cross‐validated response‐model AUCs were 0.560, 0.747, and 0.773 at Months 3, 6, and 12.
- Month 3 plasma Aβ42/40 (AUC 0.676) and p-tau217 (AUC 0.559) showed no clear superiority versus their respective baseline levels, with the change in p-tau217 yielding an AUC of 0.624.
- ARIA occurred in 13/181 participants; selective Month 12 completion limits causal interpretation.

## 1 INTRODUCTION

Alzheimer's disease (AD) is the most common cause of dementia, accounting for 60% to 70% of all dementia cases worldwide.^1^, ^2^ China faces a significant and growing challenge from AD, accounting for nearly 30% of the global burden.^3^, ^4^ AD disproportionately affects older adults, with prevalence doubling roughly every 5 years after age 65.^5^, ^6^ Beyond its devastating impact on patients and caregivers, AD imposes a substantial socioeconomic burden.^2^ Despite decades of intensive research, AD remains incurable, and disease‐modifying therapies continue to represent an urgent unmet need.^7^

The therapeutic landscape for AD has recently undergone a paradigm shift with the advent of anti‐amyloid immunotherapies.^8^ Lecanemab, a humanized monoclonal antibody targeting soluble amyloid beta (Aβ) protofibrils, received traditional US Food and Drug Administration (FDA) approval in July 2023.^9^ However, the therapy is accompanied by significant safety concerns, notably amyloid‐related imaging abnormalities (ARIA), especially in apolipoprotein E (APOE) ε4 homozygotes.^10^

The emergence of disease‐modifying therapies has simultaneously heightened the urgency for accessible, reliable biomarkers to guide diagnosis, treatment selection, and prognostic assessment.^11^, ^12^ Traditionally, confirmation of AD pathology has depended on cerebrospinal fluid (CSF) analysis and amyloid positron emission tomography (PET), both of which are invasive, costly, and largely confined to specialized centers – rendering them impractical for population‐scale screening and longitudinal monitoring.^13^ In this context, plasma biomarkers have emerged as a transformative frontier.^14^ Recently, the plasma phosphorylated tau 217 (p‐tau217)/Aβ42 ratio test (Fujirebio Lumipulse) to aid in AD diagnosis has been approved by the FDA.^15^ Our previous research using China‐developed high‐sensitivity direct chemiluminescence immunoassay also demonstrated that plasma p‐tau217 or p‐tau217/Aβ42 ratio has high diagnostic accuracy (up to 89%) for Aβ pathology.^16^

Compared with prior real‐world studies, which have predominantly emphasized short‐term safety profiles or isolated clinical endpoints,^17^, ^18^, ^19^ this investigation synthesizes multidimensional evidence encompassing longitudinal assessments applying disease‐stage‐specific meaningful within‐patient change (MWPC) thresholds at each scheduled visit and visit‐specific penalized prediction models with cross‐validated receiver operating characteristic (ROC) performance and derived exploratory predictors of treatment response. Additionally, we contrasted Month 12 completers versus non‐completers and separately analyzed early plasma biomarkers, safety outcomes, and off‐label lecanemab use in patients with moderate to severe dementia. Collectively, these analyses yield a more robust, temporally resolved, and mechanistically informed characterization of lecanemab's real‐world therapeutic profile in the Chinese population, explicitly addressing temporal dynamics, interindividual heterogeneity in treatment response, and underlying biological mechanisms.

## 2 METHODS

### 2.1 Study design and oversight

We conducted an ongoing phase 4, multicenter, prospective, real‐world study designed to evaluate the effectiveness and safety of lecanemab in participants diagnosed with early AD (i.e., mild cognitive impairment [MCI] due to AD or mild dementia due to AD), defined according to National Institute on Aging–Alzheimer's Association criteria, who met the clinical indication for lecanemab across 20 hospitals in Jiangsu Province, China.^12^, ^20^ Study sites were selected through a standardized feasibility assessment to ensure alignment with study endpoint requirements and geographic diversity. Consecutive enrollment began in June 2024, and participants received intravenous lecanemab 10 mg/kg every 2 weeks. Because enrollment was rolling, not every participant had reached each scheduled visit at the interim data cutoff of February 21, 2026.

The study was conducted in accordance with the guidelines of the International Council for Harmonisation of Technical Requirements for Pharmaceuticals for Human Use and the ethical principles outlined in the Declaration of Helsinki. Ethical approval was obtained from the Research Ethics Committee of Drum Tower Hospital (Approval No. 2024‐746‐01) and the Institutional Review Board of each participating center. Written informed consent was obtained from all participants prior to enrollment. All assessments and procedures were performed as part of routine clinical practice.

### 2.2 Participants

Key inclusion criteria were a diagnosis of early AD in individuals aged 50 to 90 years, with amyloid pathology confirmed by amyloid PET or CSF analysis, and the presence of objective impairments in episodic memory. For MCI, Mini‐Mental State Examination (MMSE) scores were required to be ≥18 for illiterate individuals, ≥21 for those with elementary education, and ≥25 for individuals with junior high school education or higher. The Clinical Dementia Rating–Global Score (CDR‐GS) for MCI was 0.5. For mild dementia, MMSE scores ranged from 14 to 17 for illiterate individuals, 14 to 20 for those with elementary education, and 20 to 24 for individuals with junior high school education or higher. The CDR‐GS for mild dementia was 1. Moderate dementia was defined by MMSE scores of 10 to 13 among illiterate and elementary‐educated individuals and 10 to 19 for those with junior high school education or above, whereas severe dementia was defined as a MMSE score below 10 across all education levels.^21^, ^22^ If a discrepancy existed between MMSE and CDR‐GS scores, the CDR‐GS took precedence. Patients with a CDR‐GS of 2 or higher were classified as moderate to severe dementia, which was beyond the approved indication for lecanemab. However, if caregivers were thoroughly briefed on the potential benefits and risks of the treatment and still chose to pursue lecanemab therapy, such patients could be enrolled in the study after providing the necessary informed consent.

**RESEARCH IN CONTEXT**

1. **Systematic review**: Randomized trials established that lecanemab slows clinical decline in early AD, but Chinese real‐world evidence on disease‐stage‐specific clinically meaningful response, follow‐up selection, and longitudinal plasma biomarkers remains limited. We reviewed lecanemab trial and real‐world literature, MWPC thresholds for CDR‐SB and iADRS, ARIA guidance, and recent plasma p‐tau assay evidence.
2. **Interpretation**: In 181 biomarker‐confirmed participants receiving biweekly lecanemab, disease‐stage‐specific CDR‐SB response increased across Months 3, 6, and 12 and was concentrated in MCI and mild dementia due to AD. Visit‐specific cross‐validated discrimination was modest to moderate. Month 3 plasma biomarkers showed only exploratory, limited discrimination of Month 12 response. Month 12 completers had less baseline cognitive impairment than participants without a Month 12 record, limiting causal interpretation. Safety findings included ARIA in 13/181 participants.
3. **Future directions**: Prospective studies should prespecify clinically meaningful response definitions, standardize visit windows and reasons for missing follow‐up, externally validate prediction models, and determine whether early plasma biomarker changes add clinically useful information beyond baseline disease stage and APOE ε4 status. Larger studies are also needed to characterize safety outside the approved early‐disease indication.

Exclusion criteria were as follows: (1) a history of transient ischemic attack, stroke, or epilepsy within 12 months prior to enrollment; (2) the presence of any psychiatric disorder or symptoms that could interfere with cognitive assessments; (3) magnetic resonance imaging (MRI) findings of 10 or more cerebral microbleeds or any single microbleed with a maximum diameter > 10 mm; (4) MRI evidence of cerebral contusion, encephalomalacia, aneurysm, vasogenic edema, or brain tumor; (5) contraindications to or intolerance of MRI examinations; (6) any uncontrolled immune‐mediated disease or a requirement for immunoglobulin therapy, systemic monoclonal antibodies, systemic immunosuppressants, or plasma exchange during the study period; and (7) uncontrolled bleeding disorders. Participants receiving oral anticoagulant therapy were required to have stable and therapeutic anticoagulation levels for at least 4 weeks prior to enrollment.

### 2.3 Protocol and clinical assessment

At baseline, participants underwent comprehensive assessments, including demographic characteristics, medical history, quality of life, cognitive status, plasma biomarkers, amyloid pathological examination, apolipoprotein E genotype, and neuroimaging indicators. Longitudinal follow‐up assessments were conducted at 3, 6, and 12 months after treatment initiation. At each visit, participants underwent safety monitoring, plasma biomarker testing, cognitive assessment, and structural neuroimaging. An additional MRI scan was performed at 1.5 months to facilitate early detection and management of ARIA. At the final 12‐month visit, participants were recommended to undergo amyloid PET or CSF testing to evaluate longitudinal changes in amyloid pathology.

Cognitive function was assessed using the MMSE, Montreal Cognitive Assessment (MoCA), and Alzheimer's Disease Assessment Scale–Cognitive Subscale 13 (ADAS‐Cog13). Daily functional ability was evaluated with the Alzheimer's Disease Cooperative Study–Activities of Daily Living (ADCS‐ADL) scale, its instrumental subscale (ADCS‐iADL), and the Functional Activities Questionnaire (FAQ). Disease severity and overall clinical progression were quantified using the CDR‐GS, the Clinical Dementia Rating–Sum of Boxes (CDR‐SB), and the Integrated Alzheimer's Disease Rating Scale (iADRS).

### 2.4 Clinically meaningful response definitions

All responder classifications were based on prespecified MWPC thresholds for CDR‐SB and iADRS. The primary MWPC threshold was a baseline reduction in CDR‐SB meeting or exceeding 0.5 points for MCI due to AD, 1 point for mild AD dementia, and 2 points for moderate to severe AD dementia.^23^, ^24^ Secondary iADRS response required an increase of at least 5 points for MCI due to AD or 9 points for mild AD dementia.^25^ No validated iADRS threshold exists for moderate to severe AD dementia, so this subgroup was excluded from secondary iADRS responder analyses.

### 2.5 PET imaging

Amyloid PET imaging was performed using the FDA‐approved radiotracer [^1^ ^8^F]‐florbetapir (AV45). Both grayscale and rainbow color scales were used for image interpretation, and results were classified dichotomously as positive or negative. In accordance with FDA‐approved criteria for [^1^ ^8^F]‐florbetapir PET, scans were deemed positive if cortical tracer uptake in one or more lobes was equal to or greater than that of adjacent white matter.^26^

### 2.6 Biomarker testing and APOE genotyping

CSF samples were obtained via standard lumbar puncture, transported on dry ice to the designated laboratory, and analyzed for biomarkers, including human Aβ42 (RE59661), Aβ40 (RE59651), total tau (RE59631), and phosphorylated tau (30121609), using enzyme‐linked immunosorbent assay.

Peripheral plasma samples were collected into ethylenediaminetetraacetic acid tubes and centrifuged at 3000 rpm for 10 min within 2 h of collection. The resulting plasma was carefully isolated, aliquoted, and immediately stored at −80°C until analysis. Plasma biomarkers were measured using the Shine i2910 Chemiluminescence Immunoassay Analyzer (IncreCare, Shenzhen, China) with Human Aβ42, Aβ40, phosphorylated tau 181 (p‐tau181), p‐tau217, neurofilament light chain, and glial fibrillary acidic protein (GFAP) detection reagents (Vazyme, Nanjing, China), following the manufacturers’ instructions.

APOE genotyping (ε2, ε3, ε4) was performed using a commercial kit from Memorigen (Xiamen, China). All assay technicians involved were blinded to the clinical data.

### 2.7 Magnetic resonance imaging

All patients underwent pretreatment (baseline) brain MRI to screen for radiological features known to affect risk for ARIA, such as microhemorrhages, superficial siderosis, infarcts, and the extent of white matter disease, as assessed using the Fazekas scale. Most baseline MRI scans were obtained in Nanjing Drum Tower Hospital on three 3.0T scanners (Philips Medical Systems, Eindhoven, The Netherlands) with T1, T2, fluid‐attenuated inversion recovery, diffusion‐weighted imaging, and susceptibility‐weighted imaging (SWI) sequences. MRI scans performed at outside facilities with different scanner strengths and sequences were reviewed by the treating clinician and two experienced neuroradiologists blinded to clinical data. For baseline MRI scans, data are presented on the original read that informed clinical decision‐making.

All follow‐up MRI scans were performed at Nanjing Drum Tower Hospital and other participating centers, typically using a 3.0T scanner. The presence and number of microhemorrhages, superficial siderosis, and ARIA with edema (ARIA‐E) were evaluated across all MRI sequences, including SWI and three‐dimensional T2‐weighted imaging. For patients who developed ARIA, data are presented on the scan with the maximum ARIA severity during the entire observation period.

### 2.8 Statistical analyses and data visualization

All statistical analyses and data visualization were conducted using Python (version 3.6.1), R (version 4.5.1), SPSS (version 26.0), and GraphPad Prism (version 8.0).

Response rates and Wilson 95% confidence intervals (CIs) were estimated at Months 3, 6, and 12 among participants with evaluable paired data. To limit overfitting, five candidate factors were selected on clinical grounds before the revised models were fitted: age, APOE ε4 status, disease stage, baseline p‐tau217, and baseline Aβ42/40. P‐tau217 and Aβ42/40 represented complementary tau and amyloid domains; disease stage was entered as two indicator variables with MCI as reference, yielding six slope parameters. Baseline MMSE was not entered alongside stage, and GFAP was not included in the multivariable model, to avoid redundant severity and biological terms. Separate exploratory Firth‐penalized logistic models were fitted at each visit; continuous predictors were standardized. Out‐of‐fold predictions were averaged across 50 repeats of five‐fold stratified cross‐validation to generate visit‐specific ROC curves and areas under the curve (AUCs). Apparent AUCs were retained in results only to show optimism.

Month 12 completers were compared with participants lacking a Month 12 record using standardized mean differences (SMDs) and exploratory tests. Among the 68 completers, baseline biomarker values, Month 3 values, and changes from baseline to Month 3 were evaluated for discrimination of Month 12 CDR‐SB response. Bootstrap 95% CIs used 10,000 resamples, and paired bootstrap differences compared Month 3 with baseline for Aβ42/40 and p‐tau217. Continuous longitudinal, subgroup, PET, and standardized response mean (SRM) analyses remained exploratory and are reported in the Supplementary Material. No multiplicity adjustment was applied.

## 3 RESULTS

### 3.1 Baseline characteristics and patient flow

The cohort included 181 participants: 59 with MCI due to AD, 77 with mild AD dementia, and 45 with moderate to severe AD dementia (41 moderate and 4 severe patients pooled due to insufficient severe subgroup sample size). Baseline characteristics are summarized in Table 1. The mean age of the cohort was 69.54 years (SD = 8.66); 61.9% were female, and 56.6% were APOE ε4 carriers. Disease stage was associated with differences in disease duration, baseline MMSE and MoCA scores, and p‐tau217 and p‐tau181 levels, whereas age, sex, and education did not differ significantly across groups.

**TABLE 1:** Baseline characteristics of study cohort stratified by disease stage.

Variable	Overall (*n* = 181)	MCI (*n* = 59)	Mild dementia (*n* = 77)	Moderate to severe dementia (*n* = 45)	*p* value
Age, years	69.54 (8.66)	69.46 (8.58)	69.75 (8.93)	69.29 (8.48)	0.935
Female, *n* (%)	112 (61.9%)	40 (67.8%)	46 (59.7%)	26 (57.8%)	0.510
Education, years	10.57 (3.99)	10.66 (4.64)	10.68 (3.89)	10.27 (3.22)	0.583
Disease duration, years	2.92 (1.79)	2.36 (1.81)	3.10 (1.87)	3.36 (1.46)	<0.001
APOE ε4 carrier, *n* (%)	98/173 (56.6%)	34/58 (58.6%)	42/73 (57.5%)	22/42 (52.4%)	0.808
Baseline MMSE	20.93 (4.98)	25.41 (2.76)	21.13 (2.39)	14.71 (3.88)	<0.001
Baseline MoCA	15.79 (5.08)	19.46 (4.54)	15.68 (3.61)	11.18 (4.02)	<0.001
Baseline Aβ42	5.53 (2.02)	5.65 (1.93)	5.66 (2.01)	5.16 (2.17)	0.179
Baseline p‐tau217	8.96 (3.56)	8.44 (3.94)	8.57 (3.46)	10.29 (2.84)	0.001
Baseline p‐tau181	7.61 (2.90)	6.93 (3.13)	7.39 (2.92)	8.89 (2.08)	<0.001
Cholinesterase inhibitor use, *n* (%)	140 (77.4%)	42 (71.2%)	60 (77.9%)	38 (84.4%)	0.274
NMDA antagonist use, *n* (%)	64 (35.4%)	12 (20.3%)	25 (32.5%)	27 (60.0%)	<0.001
Antiplatelet use, *n* (%)	29 (16.0%)	10 (16.9%)	10 (13.0%)	9 (20.0%)	0.579
Anticoagulant use, *n* (%)	4 (2.2%)	0 (0.0%)	3 (3.9%)	1 (2.2%)	0.309
Cerebral microbleeds at baseline, *n* (%)	33 (18.2%)	12 (20.3%)	16 (20.8%)	5 (11.1%)	0.360

*Note*: APOE percentages exclude eight participants with unknown genotype. Cerebral microbleeds denote at least one baseline microbleed.

Abbreviations: APOE, apolipoprotein E; Aβ, amyloid beta; MCI, mild cognitive impairment; MMSE, Mini‐Mental State Examination; MoCA, Montreal Cognitive Assessment; p‐tau, phosphorylated tau.

By the interim data cutoff of February 21, 2026, scheduled follow‐up assessments had been completed by 139 participants at Month 3, 109 at Month 6, and 68 at Month 12 (Figure 1). These counts reflect rolling follow‐up completion and should not be interpreted as 42, 72, and 113 sequential dropouts.

**FIGURE 1:**
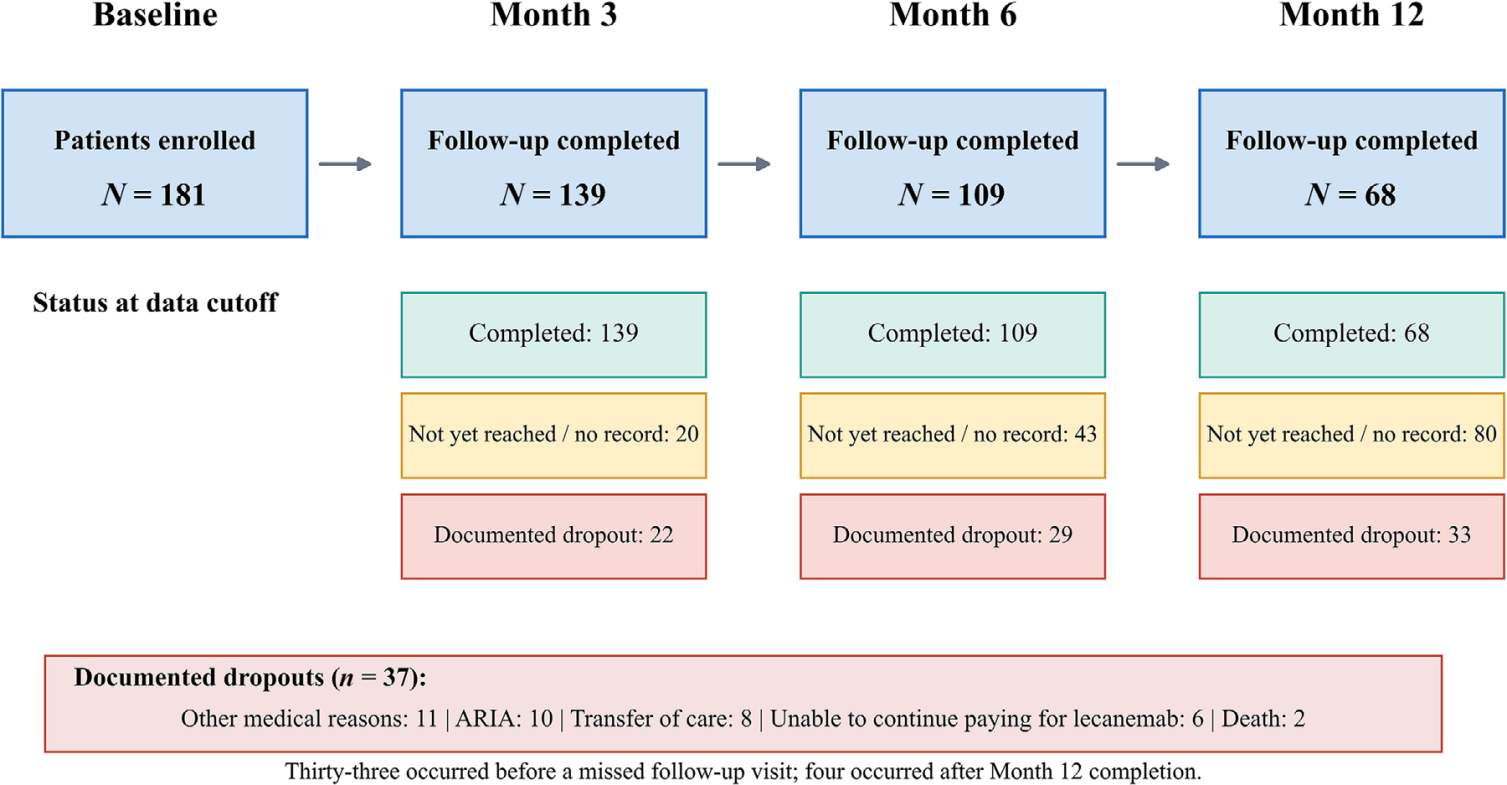
Participant follow‐up status and documented dropout. At the interim data cutoff of February 21, 2026, the diagram separates completed visits, participants who had not yet reached the visit or had no record, and documented dropout. Counts within each visit sum to the 181 enrolled participants. Thirty‐seven documented dropouts comprised other medical reasons (*n* = 11), ARIA (*n* = 10), transfer of care (*n* = 8), inability to continue paying for lecanemab (*n* = 6), and death (*n* = 2).

Thirty‐seven participants had a documented dropout reason: other medical reasons (*n* = 11), ARIA (*n* = 10), transfer of care (*n* = 8), inability to continue paying for lecanemab (*n* = 6), and death (*n* = 2). Thirty‐three discontinued before a subsequent missed visit; four completed Month 12 before later discontinuation. Site follow‐up records identified myocardial infarction (May 10, 2025) and breast cancer (January 23, 2025) as the causes of the two deaths; both were assessed as unrelated to lecanemab or ARIA.

Compared with the 113 participants without a Month 12 record, the 68 completers had higher baseline MoCA (17.21 vs 14.94; SMD 0.456; *p *= 0.004) and MMSE scores (21.93 vs 20.33; SMD 0.324; *p *= 0.039), indicating selective retention of less‐impaired participants (Table S1).

### 3.2 Disease‐stage‐specific clinically meaningful response

Of the 139, 109, and 68 participants with a recorded clinical follow‐up at Months 3, 6, and 12, paired CDR‐SB data were evaluable for 137, 106, and 68, respectively; two Month 3 and three Month 6 visits lacked a paired CDR‐SB score. The primary CDR‐SB MWPC response rate increased from 6.6% (9/137; 95% CI 3.5% to 12.0%) at Month 3 to 27.4% (29/106; 95% CI 19.8% to 36.5%) at Month 6 and 44.1% (30/68; 95% CI 32.9% to 55.9%) at Month 12 (Table 2; Figure 2A). At Month 12, response occurred in 14/28 participants with MCI (50.0%), 16/24 with mild dementia (66.7%), and 0/16 with moderate to severe dementia (0%; 95% CI 0% to 19.4%).

**TABLE 2:** Disease‐stage‐specific clinically meaningful response at Months 3, 6, and 12.

Endpoint	Visit	Group	Response (95% CI)
Primary: CDR‐SB MWPC	Month 3	Overall	9/137 (6.6%; 3.5, 12.0)
Primary: CDR‐SB MWPC	Month 3	MCI due to AD	4/47 (8.5%; 3.4, 19.9)
Primary: CDR‐SB MWPC	Month 3	Mild AD dementia	4/52 (7.7%; 3.0, 18.2)
Primary: CDR‐SB MWPC	Month 3	Moderate to severe AD dementia	1/38 (2.6%; 0.5, 13.5)
Primary: CDR‐SB MWPC	Month 6	Overall	29/106 (27.4%; 19.8, 36.5)
Primary: CDR‐SB MWPC	Month 6	MCI due to AD	17/41 (41.5%; 27.8, 56.6)
Primary: CDR‐SB MWPC	Month 6	Mild AD dementia	10/38 (26.3%; 15.0, 42.0)
Primary: CDR‐SB MWPC	Month 6	Moderate to severe AD dementia	2/27 (7.4%; 2.1, 23.4)
Primary: CDR‐SB MWPC	Month 12	Overall	30/68 (44.1%; 32.9, 55.9)
Primary: CDR‐SB MWPC	Month 12	MCI due to AD	14/28 (50.0%; 32.6, 67.4)
Primary: CDR‐SB MWPC	Month 12	Mild AD dementia	16/24 (66.7%; 46.7, 82.0)
Primary: CDR‐SB MWPC	Month 12	Moderate to severe AD dementia	0/16 (0.0%; 0.0, 19.4)
Secondary: iADRS MWPC	Month 3	Overall	1/99 (1.0%; 0.2, 5.5)
Secondary: iADRS MWPC	Month 3	MCI due to AD	1/47 (2.1%; 0.4, 11.1)
Secondary: iADRS MWPC	Month 3	Mild AD dementia	0/52 (0.0%; 0.0, 6.9)
Secondary: iADRS MWPC	Month 6	Overall	11/80 (13.8%; 7.9, 23.0)
Secondary: iADRS MWPC	Month 6	MCI due to AD	10/42 (23.8%; 13.5, 38.5)
Secondary: iADRS MWPC	Month 6	Mild AD dementia	1/38 (2.6%; 0.5, 13.5)
Secondary: iADRS MWPC	Month 12	Overall	10/52 (19.2%; 10.8, 31.9)
Secondary: iADRS MWPC	Month 12	MCI due to AD	10/28 (35.7%; 20.7, 54.2)
Secondary: iADRS MWPC	Month 12	Mild AD dementia	0/24 (0.0%; 0.0, 13.8)

*Note*: CDR‐SB thresholds: 0.5 points for MCI, 1 point for mild dementia, and 2 points for moderate to severe dementia. iADRS thresholds: 5 points for MCI and 9 points for mild dementia.

**FIGURE 2:**
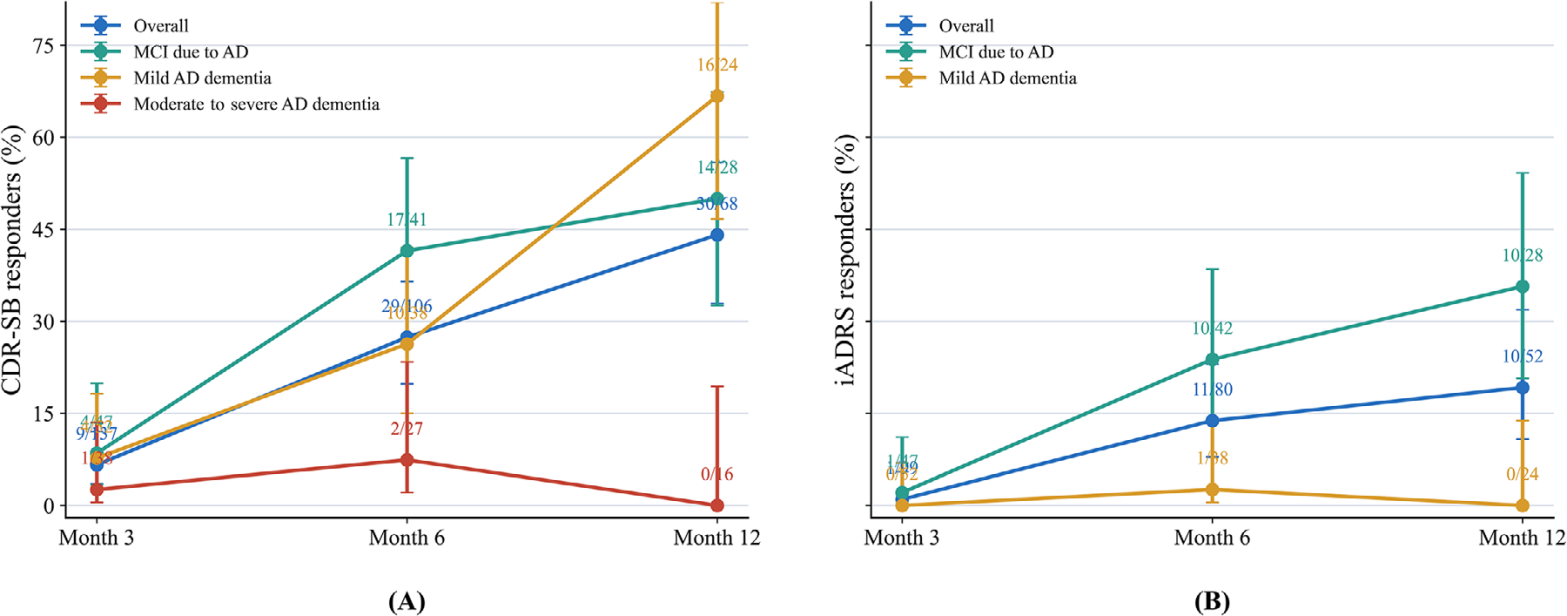
Disease‐stage‐specific clinically meaningful response over time. (A) CDR‐SB MWPC response at Months 3, 6, and 12 using stage‐specific thresholds. (B) Secondary iADRS MWPC response in MCI due to AD and mild AD dementia. Points show response rates, and error bars show Wilson 95% confidence intervals; labels show responders/evaluable participants.

Secondary iADRS response rates among participants within the approved early‐disease indication were 1.0% (1/99), 13.8% (11/80), and 19.2% (10/52) at Months 3, 6, and 12, respectively (Figure 2B). At Month 12, all 10 iADRS responders were in the MCI subgroup; no mild‐dementia participant met the 9‐point threshold.

### 3.3 Visit‐specific predictors and ROC performance

Firth models included 137, 106, and 68 participants with 9, 29, and 30 responders at Months 3, 6, and 12, respectively (Table 3). No candidate was statistically associated with response at Month 3, when events were sparse. At Month 6, higher baseline Aβ42/40 (OR 0.541, 95% CI 0.299 to 0.981; *p *= 0.043), APOE ε4 carriage (OR 0.169, 95% CI 0.060 to 0.479; *p *< 0.001), and moderate to severe dementia versus MCI (OR 0.041, 95% CI 0.006 to 0.265; *p *< 0.001) were associated with lower response odds. At Month 12, APOE ε4 carriage (OR 0.213, 95% CI 0.059 to 0.764; *p *= 0.018) and moderate to severe dementia versus MCI (OR 0.018, 95% CI 0.001 to 0.380; *p *= 0.010) were associated with lower response odds. All estimates are exploratory.

**TABLE 3:** Firth‐penalized logistic regression of CDR‐SB response by visit.

Visit	Predictor	Scale	OR (95% CI)	*P*	Model N	Responders	Repeated CV AUC	Apparent AUC
Month 3	Age	Per SD	0.745 (0.403, 1.379)	0.349	137	9	0.560	0.753
Month 3	Baseline p‐tau217	Per SD	0.578 (0.26, 1.279)	0.176	137	9	0.560	0.753
Month 3	Baseline Aβ42/40	Per SD	0.733 (0.34, 1.540)	0.412	137	9	0.560	0.753
Month 3	APOE ε4 carrier	Binary contrast	0.615 (0.177, 2.135)	0.444	137	9	0.560	0.753
Month 3	Mild dementia versus MCI	Binary contrast	0.903 (0.235, 3.470)	0.882	137	9	0.560	0.753
Month 3	Moderate to severe dementia vs MCI	Binary contrast	0.399 (0.053, 2.990)	0.372	137	9	0.560	0.753
Month 6	Age	Per SD	0.977 (0.597, 1.601)	0.927	106	29	0.747	0.823
Month 6	Baseline p‐tau217	Per SD	1.012 (0.615, 1.665)	0.963	106	29	0.747	0.823
Month 6	Baseline Aβ42/40	Per SD	0.541 (0.299, 0.981)	0.043	106	29	0.747	0.823
Month 6	APOE ε4 carrier	Binary contrast	0.169 (0.060, 0.479)	<0.001	106	29	0.747	0.823
Month 6	Mild dementia versus MCI	Binary contrast	0.453 (0.157, 1.308)	0.143	106	29	0.747	0.823
Month 6	Moderate to severe dementia versus MCI	Binary contrast	0.041 (0.006, 0.265)	<0.001	106	29	0.747	0.823
Month 12	Age	Per SD	1.269 (0.672, 2.394)	0.463	68	30	0.773	0.855
Month 12	Baseline p‐tau217	Per SD	1.040 (0.569, 1.900)	0.899	68	30	0.773	0.855
Month 12	Baseline Aβ42/40	Per SD	0.809 (0.435, 1.502)	0.502	68	30	0.773	0.855
Month 12	APOE ε4 carrier	Binary contrast	0.213 (0.059, 0.764)	0.018	68	30	0.773	0.855
Month 12	Mild dementia versus MCI	Binary contrast	2.045 (0.573, 7.307)	0.271	68	30	0.773	0.855
Month 12	Moderate to severe dementia versus MCI	Binary contrast	0.018 (0.001, 0.380)	0.010	68	30	0.773	0.855

*Note*: Continuous predictors were standardized. Disease stage was entered as two indicator variables with MCI as reference; therefore, five conceptual predictors yielded six slope parameters. Repeated CV AUC denotes AUC from out‐of‐fold predictions averaged across 50 repeats of five‐fold stratified cross‐validation. Apparent AUC is shown only to demonstrate optimism. All participants with evaluable CDR‐SB response had complete data for the selected model predictors.

Repeated five‐fold cross‐validated AUCs were 0.560 at Month 3, 0.747 at Month 6, and 0.773 at Month 12 (Figure 3). These were lower than apparent AUCs, especially at Month 3, and are reported as exploratory internal estimates rather than validated prognostic performance.

**FIGURE 3:**
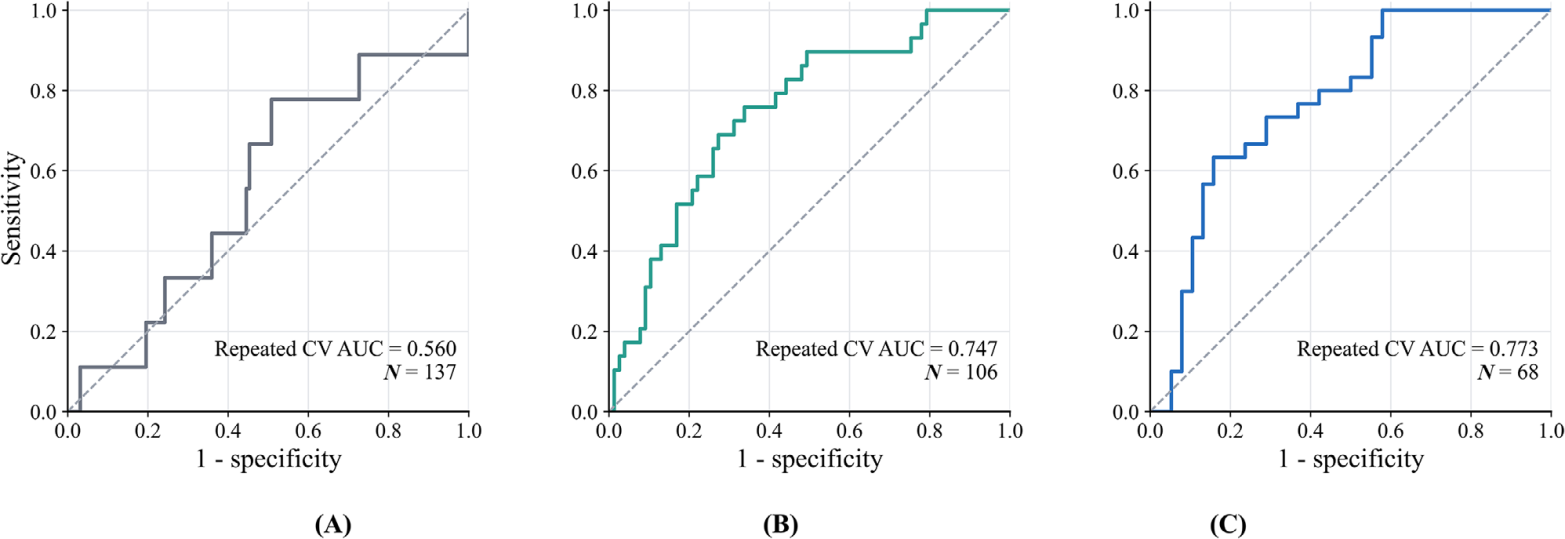
Cross‐validated ROC performance of visit‐specific CDR‐SB response models. Panels show ROC curves based on out‐of‐fold predictions averaged across 50 repeats of five‐fold stratified cross‐validation at Month 3 (A), Month 6 (B), and Month 12 (C). The diagonal line indicates chance discrimination.

### 3.4 Early on‐treatment plasma biomarkers for predicting clinical response

Among 68 Month 12 completers, baseline values were included as comparators for the early on‐treatment analyses. Month 3 Aβ42/40 had an AUC of 0.676 (95% CI 0.539 to 0.808), compared with 0.562 (95% CI 0.422 to 0.699) at baseline; the paired AUC difference was 0.114 (95% CI −0.042 to 0.269). P‐tau217 had similar AUCs at baseline (0.551) and Month 3 (0.559), with a paired difference of 0.007 (95% CI −0.105 to 0.111), whereas its change from baseline to Month 3 yielded an AUC of 0.624 (95% CI 0.485 to 0.755). Most CIs included 0.5, and the paired comparisons did not establish superiority of Month 3 values over baseline (Table S2; Figure 4).

**FIGURE 4:**
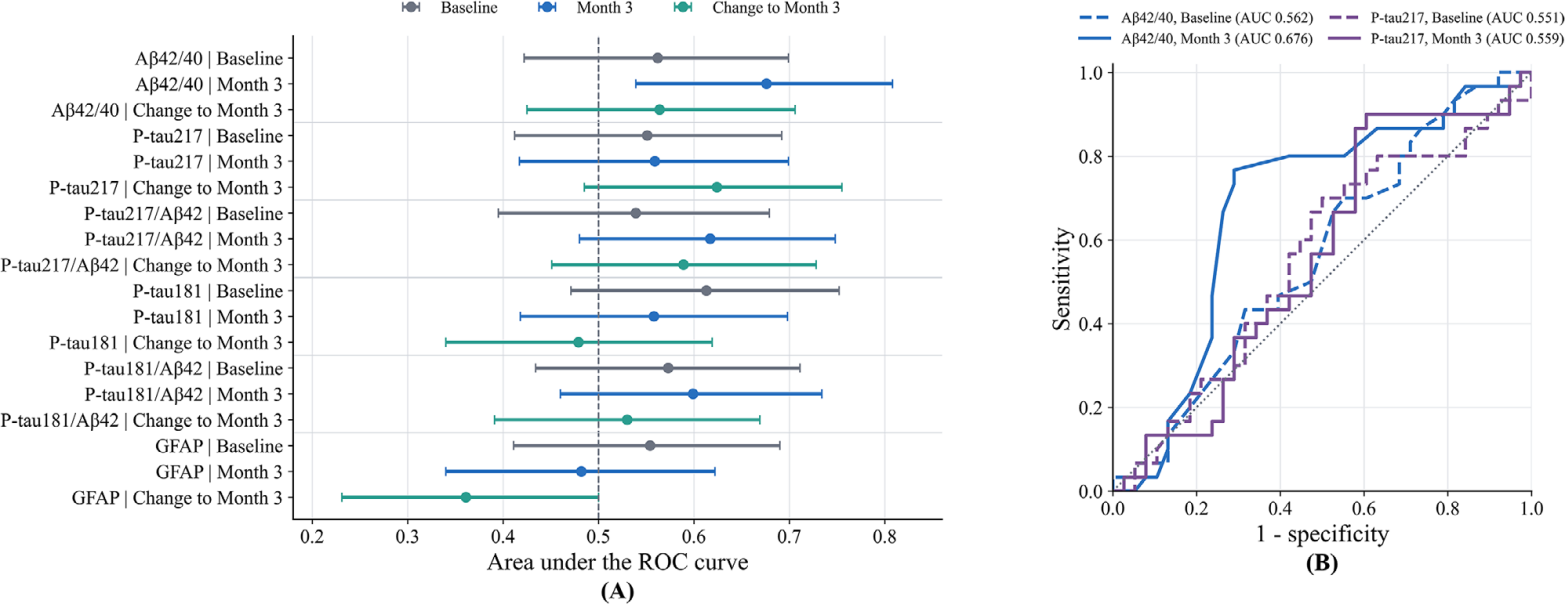
Baseline and Month 3 plasma biomarkers and Month 12 CDR‐SB response. (A) AUCs and bootstrap 95% confidence intervals for baseline values, Month 3 values, and changes from baseline to Month 3 among 68 Month 12 completers. (B) Baseline and Month 3 ROC curves for Aβ42/40 and p‐tau217. For biomarkers expected to decrease with response, values were direction‐coded so that a higher score indicated response. Analyses were exploratory.

### 3.5 Continuous outcomes, amyloid PET, and subgroup analyses

Continuous analyses remained supportive and are presented in Tables S3 to S7 and Figures S1 to S3. Clinical changes were generally more apparent at Months 6 and 12, whereas several plasma biomarkers changed by Month 3. Among the clinical and biomarker outcomes, p‐tau217 showed the largest absolute SRM at Month 12 (SRM = −1.23), indicating the greatest standardized longitudinal change. In 27 participants with paired amyloid PET scans, global SUVR decreased from 1.74 (SD 0.34) to 1.35 (SD 0.22; mean change −0.38, *p *< 0.001).

In the moderate‐to‐severe subgroup, exploratory continuous changes suggested signals in selected scales and biomarkers (Figure S4), but clinically meaningful CDR‐SB response remained uncommon (1/38 at Month 3, 2/27 at Month 6, and 0/16 at Month 12; Figure 5A). Thus, mean change should not be interpreted as evidence that individual patients met the prespecified MWPC threshold.

**FIGURE 5:**
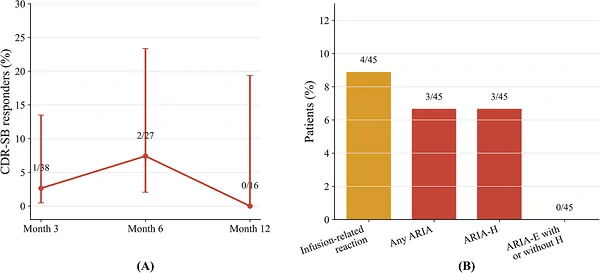
Clinically meaningful response and safety in moderate to severe AD dementia. (A) Stage‐specific CDR‐SB MWPC response rates at Months 3, 6, and 12. (B) Infusion‐related reactions and ARIA. Error bars show Wilson 95% confidence intervals for response rates; labels show events/evaluable participants.

### 3.6 Safety

Infusion‐related reactions occurred in 12/181 participants (6.6%). ARIA occurred in 13/181 participants (7.2%), all of which were asymptomatic: 10 had isolated ARIA with hemorrhage (ARIA‐H), and three had ARIA‐E with or without concurrent ARIA‐H. ARIA was recorded in 10/37 documented dropouts (27.0%), compared with 3/144 participants without documented dropout. In moderate to severe dementia, infusion reactions occurred in 4/45 (8.9%) and ARIA in 3/45 (6.7%), all ARIA‐H (Table S8; Figure 5B and Figure S5).

## 4 DISCUSSION

In a multicenter real‐world cohort study conducted in Jiangsu, China, we evaluated the effects of lecanemab treatment in patients with AD. Our key findings are as follows: (1) Using disease‐stage‐specific MWPC thresholds as the primary framework, clinical response rates rose progressively from Month 3 to Month 12; robust cognitive benefits were restricted to MCI and mild AD dementia, while no participants with moderate to severe AD achieved CDR‐SB‐defined clinical improvement at Month 12. (2) Patients with milder disease or without APOE ε4 showed greater treatment benefits at 6 to 12 months. (3) Lecanemab improved plasma biomarkers (p‐tau217, Aβ42/40, p‐tau217/Aβ42) as early as Month 3, preceding overt cognitive improvements at Months 6 to 12. (4) Among 68 patients completing 12 months of treatment, Month 3 Aβ42/40 exhibited the strongest discriminative capacity for identifying patients with a clinically meaningful response according to ROC AUC analyses. (5) Moderate to severe dementia patients achieved milder benefits in biomarkers and symptoms, with good safety.

As of the data cutoff date, 144 patients remained engaged in follow‐up, while only 68 completed the full 12‐month treatment assessment. Conclusions regarding the long‐term efficacy of lecanemab remain preliminary and will require validation using complete, long‐term follow‐up data. Notably, although only 109 patients had completed 6‐month follow‐up at the time of this interim analysis, the available data already indicate a positive therapeutic signal.

Consistent with previously reported studies,^9^, ^20^, ^27^ lecanemab therapy improves cognitive function in patients with AD after 6 to 12 months of treatment, particularly in those with MCI and mild dementia. Our study further revealed that clinically meaningful improvements meeting prespecified MWPC thresholds were rare at Month 3, with tangible therapeutic benefits beginning to emerge after Month 6 and becoming more pronounced at Month 12. This temporal pattern was quantified using primary CDR‐SB responder analyses, which identified progressively rising response rates of 6.6% at Month 3, 27.4% at Month 6, and 44.1% at Month 12, illustrating delayed clinical benefit from continuous anti‐amyloid therapy. Restricted to approved early AD patients, secondary iADRS analyses reproduced this upward trend over time. Notably, all participants meeting the predefined 9‐point iADRS improvement threshold at Month 12 had MCI, whereas no patients with mild AD dementia achieved this cutoff.

Stratified subgroup analyses revealed stark disparities in therapeutic gains across AD severity stages. Half of MCI patients (50.0%) and two‐thirds of mild AD dementia patients (66.7%) attained CDR‐SB‐based clinically meaningful response at Month 12, whereas zero participants with moderate to severe AD dementia achieved individual‐level clinical improvement despite modest aggregate shifts on continuous cognitive and functional scales.

Factors predicting clinically meaningful response to lecanemab therapy were also explored in our study. Covariate‐adjusted Firth logistic regression revealed that APOE ε4 non‐carriers and patients at earlier disease stages were more likely to experience clinical improvement. Elevated baseline Aβ42/40 independently favored better response only at Month 6. Age, sex, education, disease duration, baseline MMSE, p‐tau217, and GFAP were not significantly associated with improvement. The cross‐validated prognostic model exhibited moderate discrimination for 12‐month clinical response (AUC = 0.773), with weaker predictive performance at Month 3 (AUC = 0.560).

Another distinctive feature of our study is the evaluation of early plasma biomarkers for predicting lecanemab therapeutic response. Plasma Aβ42/40 and p‐tau217 changed significantly as early as Month 3, prior to clinical cognitive improvements at Months 6 to 12. Among all tested indicators, Month 3 Aβ42/40 showed the best ability to discriminate patients with 12‐month clinically meaningful response (AUC = 0.676). Changes in p‐tau217 only offered weak to moderate predictive power.

Multiple complementary cognitive and functional instruments, including MMSE, MoCA, ADAS‐Cog13, ADCS‐ADL, ADCS‐iADL, iADRS, CDR‐SB, and FAQ, corroborated these stage‐specific therapeutic effects. Consistent with phase 3 trial findings, larger longitudinal improvements were observed on ADCS‐ADL relative to CDR‐SB at Month 6.^9^ Critically, small mean‐level biomarker and scale fluctuations observed in the moderate to severe cohort cannot be interpreted as evidence of individual clinical benefit, a finding that urges caution regarding off‐label lecanemab use in advanced AD dementia.

Plasma biomarkers exhibited longitudinal changes throughout treatment, with p‐tau217 demonstrating the largest absolute SRM at Month 12. Combined with the observed reduction in amyloid PET SUVR, these results support p‐tau217 as an accessible pharmacodynamic biomarker to track treatment response, providing cost‐effective, convenient surrogate measures for therapeutic evaluation.

In a subset of patients who completed 12 months of treatment, 33.33% achieved amyloid PET‐negative status. In real‐world cohorts, amyloid PET‐negative conversion rates of 24.1% and 29.2% have been reported at 6 months,^17^, ^19^ whereas corresponding 12‐month data remain limited. In the phase 3 CLARITY‐AD study, higher conversion rates were observed, reaching 36% at 6 months and 54% at 12 months.^9^

Notably, patients with moderate to severe AD exhibited measurable reductions in amyloid pathology as indicated by plasma biomarkers, yet few of these individuals achieved MWPC‐based clinical improvement, demonstrating a clear dissociation between pathological alterations and cognitive clinical outcomes in advanced AD.

Lecanemab was generally well tolerated in this study. Infusion‐related reactions occurred in 12/181 participants (6.63%), with fever being the most common. ARIA was observed in 13/181 participants (7.18%), predominantly ARIA‐H, and all cases were asymptomatic. Adverse event rates were comparable to or lower than those reported in clinical trials and prior real‐world studies. A prospective real‐world study reported an ARIA rate of 12.1%, mainly ARIA‐H and asymptomatic.^28^ Similarly, a large cohort found that ARIA was largely asymptomatic, with ARIA‐H occurring more frequently than ARIA‐E during lecanemab treatment.^29^

Among treatment‐related causes of discontinuation, ARIA was the predominant driver, followed by financial constraints. The relatively lower incidence of ARIA and infusion reactions may be attributed to cautious patient selection, a lower proportion of APOE ε4 carriers, and reduced use of anticoagulants.

The Chinese‐approved indication for lecanemab is limited to MCI due to AD and mild AD dementia. After 18 months of treatment with 10 mg/kg every 2 weeks, the NMPA‐approved labeling allows either continued biweekly dosing or a switch to 10 mg/kg every 4 weeks.^30^ Progression beyond the approved indication therefore makes continued treatment off‐label and requires an individualized benefit‐risk reassessment, including clinical trajectory, ARIA findings, treatment goals, and patient and caregiver preferences. In ongoing care, treating clinicians must plan to reassess observed benefits with patients and caregivers at the 6‐ and 12‐month visits and to consider discontinuation after shared decision‐making when benefit is judged insufficient. MRI‐guided interruption or discontinuation for ARIA should follow its clinical and radiographic severity and appropriate‐use guidance.^20^, ^30^

## 5 LIMITATIONS

Several limitations exist. First, the ongoing rolling cohort had only 68 patients complete 12 months of treatment, limiting long‐term evaluation. Second, the single‐arm design lacked a matched untreated control. Third, sparse clinical response events at Month 3 and in the moderate‐to‐severe AD subgroup reduced the statistical precision of multivariable analyses. Fourth, subgroup and exploratory analyses were not adjusted for multiple comparisons.

## 6 CONCLUSION

Lecanemab therapy demonstrates efficacy and safety in the treatment of AD over a period of 6 to 12 months. Patients with early‐stage AD and those who are APOE ε4 non‐carriers may derive greater benefit, whereas patients with moderate to severe dementia may experience only limited therapeutic gains. Changes in plasma biomarkers precede clinical improvements during lecanemab treatment, and p‐tau217 can function as a dynamic biomarker for tracking treatment response.

## CONFLICT OF INTEREST STATEMENT

The authors declare no conflicts of interest. Author disclosures are available in the Supporting Information.

## CONSENT STATEMENT

All participants provided written informed consent prior to their involvement in the study. The study protocol was reviewed and approved by the Ethics Committees of all participating medical centers (ethical approval number 2024‐746‐01).

## Supporting information



Supporting Information

Supporting Information
