# Supplementary material for: Real‐world clinical, biomarker, and imaging outcomes of lecanemab in Alzheimer's disease: a multicenter longitudinal cohort study in Jiangsu, China
Source: Alzheimers Dement. 2026 Oct 26;22(10):e71936. doi: 10.1002/alz.71936 (PMC13710672; doi:10.1002/alz.71936)
Supplement: Supplementary file 2 [file ALZ-22-e71936-s002.docx]

**Journal**: Alzheimer's & dementia

**Article title:** Real-World Clinical, Biomarker, and Imaging Outcomes of Lecanemab in Alzheimer's Disease: A Multicentre Longitudinal Cohort Study in Jiangsu, China

**Author names**: Limoran Tang^1#^, Xicheng Chen^6#^, Li Zhang^7^, Lin Fan^8^, Nihong Chen^9^, Chaosheng Li^10^, Zenglin Cai^11^, Guofang Chen^12^, Haifeng Chen^1^, Min Ye^13^, Huifen Zheng^14^, Qi Li^1^, Zhihong Ke^1^, Zheqi Hu^1^, Maohong Cao^15,^ Dan Yang^1^, Shuai Xu^1^, Chunming Xie^16^, Xiaobo Li^17^, Xiaotian Xu^18^, Ming Yu^19^, Xuegan Lian^20^, Hua Hu^21^, Hongfei Li^22^, Xiuying Cai^23^, Hong Chen^24^, Wu Xu^25^, Guiyun Cui^26^, Yang Zhao^6^, Jingde Dong^7^, Lili Zhang^10^, Xiaolei Zhu^1,2,3,4,5*^, Hui Zhao^1*^, Yun Xu^1,2,3,4,5*^

^#^Limoran Tang and Xicheng Chen have contributed equally to this work and share first authorship.

^*^ These authors are co-corresponding authors.

**Corresponding author affiliation and e-mail address**:

Yun Xu (Principal corresponding Author), Department of Neurology, Affiliated Drum Tower Hospital of Nanjing University Medical School; 321 ZhongShan Road, Nanjing, Jiangsu, 210008, China. Fax: +86-25-83105208; Tel: +86-25-68182212; Telephone: +86-13914764479; E-mail: xuyun20042001@aliyun.com

Hui Zhao (Co-corresponding Author), Department of Neurology, Affiliated Drum Tower Hospital of Nanjing University Medical School; 321 ZhongShan Road, Nanjing, Jiangsu, 210008, China. Fax: +86-25-83105208; Tel: +86-25-68182212; Telephone: +86-13605140127; E-mail: zhaohuigyc2002@163.com

Xiaolei Zhu (Co-corresponding Author), Department of Neurology, Affiliated Drum Tower Hospital of Nanjing University Medical School; 321 ZhongShan Road, Nanjing, Jiangsu, 210008, China. Fax: +86-25-83105208; Tel: +86-25-68182212; Telephone: +86-13851529145; E-mail: zhuquelee@126.com

Supplementary Material

**Table S1. Baseline comparison of Month 12 completers and participants without a Month 12 record.**

| **Variable** | **Month 12 completers (n=68)** | **No Month 12 data (n=113)** | **SMD** | **P value display** |
| --- | --- | --- | --- | --- |
| Age, years | 68.66 (9.19) | 70.07 (8.31) | -0.163 | 0.303 |
| Male sex | 26 (38.2%) | 43 (38.1%) | 0.004 | 0.98 |
| Education, years | 11.16 (3.56) | 10.21 (4.20) | 0.239 | 0.106 |
| Disease duration, years | 2.78 (1.94) | 3.01 (1.70) | -0.131 | 0.409 |
| APOE e4 carrier | 33 (48.5%) | 65 (61.9%) | -0.271 | 0.083 |
| MCI due to AD | 28 (41.2%) | 31 (27.4%) | 0.293 | 0.056 |
| Mild AD dementia | 24 (35.3%) | 53 (46.9%) | -0.238 | 0.126 |
| Moderate-to-severe AD dementia | 16 (23.5%) | 29 (25.7%) | -0.05 | 0.748 |
| Baseline MMSE | 21.93 (5.13) | 20.33 (4.81) | 0.324 | 0.039 |
| Baseline MoCA | 17.21 (5.00) | 14.94 (4.96) | 0.456 | 0.004 |
| Baseline ADAS-Cog13 | 24.57 (12.57) | 26.10 (11.78) | -0.126 | 0.42 |
| Baseline ADCS-ADL | 59.00 (15.17) | 56.83 (14.13) | 0.149 | 0.341 |
| Baseline ADCS-iADL | 40.46 (12.65) | 38.94 (12.36) | 0.122 | 0.432 |
| Baseline iADRS | 100.88 (24.71) | 97.84 (23.55) | 0.127 | 0.416 |
| Baseline CDR-SB | 4.44 (3.92) | 4.77 (3.56) | -0.089 | 0.573 |
| Baseline CDR-GS | 0.99 (0.67) | 1.07 (0.68) | -0.115 | 0.453 |
| Baseline FAQ | 9.29 (5.11) | 9.87 (4.82) | -0.116 | 0.457 |
| Baseline P-tau217 | 8.89 (2.94) | 8.99 (3.89) | -0.028 | 0.847 |
| Baseline P-tau181 | 7.82 (2.50) | 7.49 (3.12) | 0.115 | 0.432 |
| Baseline Aβ42 | 5.77 (1.85) | 5.39 (2.12) | 0.191 | 0.201 |
| Baseline Aβ40 | 134.20 (32.28) | 136.11 (55.70) | -0.04 | 0.77 |
| Baseline Aβ42/40 | 0.04 (0.01) | 0.04 (0.02) | 0.106 | 0.477 |
| Baseline P-tau217/Aβ42 | 1.91 (1.95) | 2.14 (1.88) | -0.117 | 0.453 |
| Baseline P-tau181/Aβ42 | 1.70 (1.72) | 1.78 (1.60) | -0.043 | 0.782 |
| Baseline GFAP | 198.09 (104.42) | 210.01 (122.55) | -0.103 | 0.489 |
| Baseline NfL | 70.90 (67.71) | 105.93 (171.83) | -0.248 | 0.059 |
| Cholinesterase inhibitor use | 56 (82.4%) | 84 (74.3%) | 0.196 | 0.212 |
| NMDA antagonist use | 26 (38.2%) | 38 (33.6%) | 0.096 | 0.53 |
| Antiplatelet use | 10 (14.7%) | 19 (16.8%) | -0.058 | 0.708 |
| Anticoagulant use | 3 (4.4%) | 1 (0.9%) | 0.221 | 0.15 |
| Any baseline microbleed | 11 (16.2%) | 22 (19.5%) | -0.086 | 0.578 |
| Baseline Fazekas score | 1.57 (0.87) | 1.65 (0.98) | -0.077 | 0.606 |
| Baseline iron deposition | 0 (0.0%) | 3 (2.7%) | -0.234 | 0.292 |

SMD, standardized mean difference. APOE carrier percentages exclude unknown genotype. Any baseline microbleed denotes at least one microbleed. P values are exploratory and were not adjusted for multiplicity.

**Table S2. Baseline and Month 3 plasma biomarkers for discrimination of Month 12 CDR-SB response.**

| **Biomarker** | **Feature** | **N** | **Responders** | **AUC (95% CI)** | **Direction coded as higher response score** |
| --- | --- | --- | --- | --- | --- |
| Aβ42/40 | Baseline value | 68 | 30 | 0.562 (0.422-0.699) | higher |
| Aβ42/40 | Month 3 value | 68 | 30 | 0.676 (0.539-0.808) | higher |
| Aβ42/40 | Change from baseline to Month 3 | 68 | 30 | 0.564 (0.425-0.706) | higher |
| P-tau217 | Baseline value | 68 | 30 | 0.551 (0.412-0.692) | lower |
| P-tau217 | Month 3 value | 68 | 30 | 0.559 (0.417-0.699) | lower |
| P-tau217 | Change from baseline to Month 3 | 68 | 30 | 0.624 (0.485-0.755) | lower |
| P-tau217/Aβ42 | Baseline value | 68 | 30 | 0.539 (0.395-0.679) | lower |
| P-tau217/Aβ42 | Month 3 value | 68 | 30 | 0.617 (0.480-0.748) | lower |
| P-tau217/Aβ42 | Change from baseline to Month 3 | 68 | 30 | 0.589 (0.451-0.728) | lower |
| P-tau181 | Baseline value | 68 | 30 | 0.613 (0.471-0.752) | lower |
| P-tau181 | Month 3 value | 68 | 30 | 0.558 (0.418-0.698) | lower |
| P-tau181 | Change from baseline to Month 3 | 68 | 30 | 0.479 (0.340-0.619) | lower |
| P-tau181/Aβ42 | Baseline value | 68 | 30 | 0.573 (0.434-0.711) | lower |
| P-tau181/Aβ42 | Month 3 value | 68 | 30 | 0.599 (0.460-0.734) | lower |
| P-tau181/Aβ42 | Change from baseline to Month 3 | 68 | 30 | 0.530 (0.391-0.669) | lower |
| GFAP | Baseline value | 68 | 30 | 0.554 (0.411-0.690) | lower |
| GFAP | Month 3 value | 68 | 30 | 0.482 (0.340-0.622) | lower |
| GFAP | Change from baseline to Month 3 | 68 | 30 | 0.361 (0.231-0.500) | lower |

AUC confidence intervals were obtained from 10,000 bootstrap replicates. Baseline values are shown as comparators for the early on-treatment Month 3 analyses. The paired Month 3-minus-baseline AUC differences were 0.114 (95% CI -0.042 to 0.269) for Aβ42/40 and 0.007 (95% CI -0.105 to 0.111) for P-tau217. Analyses were exploratory among the 68 Month 12 completers.

| **Table S3. Changes in clinical scales at Months 3, 6, and 12.** | | | | | | | |  |
| --- | --- | --- | --- | --- | --- | --- | --- | --- |
| **Clinical scale** | **Visit (M)** | **N** | **Baseline** | **Follow-up** | **Mean change** | **95% CI** | ***P* value** |  |
| MMSE | 3 | 139 | 21.19 (5.00) | 21.20 (4.82) | 0.01 | (-0.25, 0.26) | 0.956 |  |
| MoCA | 3 | 139 | 16.03 (5.25) | 16.00 (5.00) | -0.03 | (-0.28, 0.22) | 0.820 |  |
| ADAS-Cog13 | 3 | 135 | 25.36 (12.44) | 25.35 (12.35) | -0.01 | (-0.21, 0.20) | 0.943 |  |
| ADCS-ADL | 3 | 135 | 57.51 (14.89) | 57.64 (14.69) | 0.13 | (-0.21, 0.48) | 0.446 |  |
| ADCS-iADL | 3 | 135 | 39.47 (12.88) | 39.70 (12.78) | 0.23 | (-0.12, 0.58) | 0.199 |  |
| iADRS | 3 | 135 | 99.11 (24.77) | 99.35 (24.58) | 0.24 | (-0.30, 0.77) | 0.388 |  |
| CDR-SB | 3 | 137 | 4.65 (3.70) | 4.59 (3.67) | -0.06 | (-0.15, 0.04) | 0.229 |  |
| FAQ | 3 | 135 | 9.56 (5.06) | 9.53 (5.05) | -0.04 | (-0.12, 0.04) | 0.371 |  |
| MMSE | 6 | 109 | 21.53 (4.87) | 21.63 (5.27) | 0.10 | (-0.12, 0.33) | 0.379 |  |
| MoCA | 6 | 109 | 16.39 (5.26) | 16.62 (5.28) | 0.24 | (-0.11, 0.58) | 0.178 |  |
| ADAS-Cog13 | 6 | 106 | 26.05 (13.13) | 25.30 (13.39) | -0.75 | (-1.01, -0.48) | <0.001 |  |
| ADCS-ADL | 6 | 106 | 57.09 (15.32) | 57.95 (15.92) | 0.86 | (0.56, 1.16) | <0.001 |  |
| ADCS-iADL | 6 | 107 | 39.09 (13.21) | 39.75 (14.41) | 0.65 | (-0.36, 1.67) | 0.208 |  |
| iADRS | 6 | 107 | 98.18 (25.78) | 99.68 (26.78) | 1.51 | (0.62, 2.39) | 0.001 |  |
| CDR-SB | 6 | 106 | 4.74 (3.86) | 4.46 (3.91) | -0.27 | (-0.40, -0.15) | <0.001 |  |
| FAQ | 6 | 106 | 9.64 (5.12) | 9.19 (5.31) | -0.45 | (-0.65, -0.25) | <0.001 |  |
| MMSE | 12 | 68 | 21.93 (5.13) | 22.50 (6.19) | 0.57 | (0.09, 1.06) | 0.022 |  |
| MoCA | 12 | 68 | 17.21 (5.00) | 17.56 (4.77) | 0.35 | (-0.11, 0.82) | 0.142 |  |
| ADAS-Cog13 | 12 | 68 | 24.57 (12.57) | 23.63 (13.21) | -0.94 | (-1.47, -0.42) | <0.001 |  |
| ADCS-ADL | 12 | 68 | 59.00 (15.17) | 59.85 (16.62) | 0.85 | (0.25, 1.46) | 0.007 |  |
| ADCS-iADL | 12 | 68 | 40.46 (12.65) | 43.04 (13.12) | 2.59 | (1.97, 3.21) | <0.001 |  |
| iADRS | 12 | 68 | 100.88 (24.71) | 104.37 (25.24) | 3.49 | (2.64, 4.33) | <0.001 |  |
| CDR-SB | 12 | 68 | 4.44 (3.92) | 3.97 (4.07) | -0.47 | (-0.65, -0.29) | <0.001 |  |
| FAQ | 12 | 68 | 9.29 (5.11) | 8.09 (5.31) | -1.21 | (-1.61, -0.80) | <0.001 |  |
| Abbreviations: ADAS-Cog13, 13-item cognitive subscale of the Alzheimer's Disease Assessment Scale; ADCS-ADL, Alzheimer's Disease Cooperative Study—Activities of Daily Living; ADCS-iADL, Alzheimer's Disease Cooperative Study—Instrumental Activities of Daily Living; CDR-SB, sum of boxes of the Clinical Dementia Rating Scale; CI, confidence interval; FAQ, Functional Activities Questionnaire; iADRS, Integrated Alzheimer's Disease Rating Scale; MMSE, Mini-Mental State Examination; MoCA, The Montreal Cognitive Assessment. | | | | | | | |  |
|  |  |  |  |  |  |  |  |  |
|  |  |  |  |  |  |  |  |  |
|  |  |  |  |  |  |  |  |  |
|  |  |  |  |  |  |  |  |  |

| **Table S4. Changes in plasma biomarkers at Months 3, 6, and 12.** | | | | | | | |  |
| --- | --- | --- | --- | --- | --- | --- | --- | --- |
| **Biomarker** | **Visit (M)** | **N** | **Baseline** | **Follow-up** | **Mean change** | **95% CI** | ***P* value** |  |
| P-tau217 | 3 | 139 | 9.02 (3.52) | 8.20 (3.97) | -0.81 | (-1.26, -0.36) | <0.001 |  |
| P-tau181 | 3 | 138 | 7.70 (2.94) | 7.12 (3.33) | -0.58 | (-0.98, -0.19) | 0.005 |  |
| Aβ42 | 3 | 139 | 5.38 (1.99) | 5.80 (2.09) | 0.41 | (0.10, 0.72) | 0.010 |  |
| Aβ40 | 3 | 139 | 131.82 (35.90) | 132.20 (29.97) | 0.37 | (-5.01, 5.76) | 0.892 |  |
| Aβ42/40 | 3 | 139 | 0.04 (0.01) | 0.04 (0.01) | 0.00 | (0.00, 0.01) | 0.011 |  |
| P-tau217/Aβ42 | 3 | 139 | 2.18 (2.06) | 1.73 (1.35) | -0.45 | (-0.73, -0.17) | 0.002 |  |
| P-tau181/Aβ42 | 3 | 138 | 1.86 (1.79) | 1.49 (1.09) | -0.37 | (-0.61, -0.13) | 0.003 |  |
| GFAP | 3 | 137 | 198.95 (110.26) | 175.15 (85.01) | -23.81 | (-37.55, -10.06) | <0.001 |  |
| NfL | 3 | 137 | 76.72 (64.47) | 73.68 (68.71) | -3.04 | (-7.79, 1.72) | 0.213 |  |
| P-tau217 | 6 | 102 | 9.25 (3.01) | 6.86 (3.58) | -2.39 | (-2.89, -1.89) | <0.001 |  |
| P-tau181 | 6 | 101 | 8.02 (2.51) | 6.63 (3.10) | -1.39 | (-1.82, -0.96) | <0.001 |  |
| Aβ42 | 6 | 102 | 5.49 (2.05) | 6.23 (1.75) | 0.74 | (0.33, 1.15) | <0.001 |  |
| Aβ40 | 6 | 102 | 136.06 (36.58) | 129.09 (31.11) | -6.97 | (-14.07, 0.13) | 0.057 |  |
| Aβ42/40 | 6 | 102 | 0.04 (0.01) | 0.05 (0.01) | 0.01 | (0.01, 0.01) | <0.001 |  |
| P-tau217/Aβ42 | 6 | 102 | 2.18 (2.01) | 1.26 (1.01) | -0.92 | (-1.30, -0.54) | <0.001 |  |
| P-tau181/Aβ42 | 6 | 101 | 1.91 (1.82) | 1.21 (0.86) | -0.69 | (-1.03, -0.36) | <0.001 |  |
| GFAP | 6 | 102 | 196.58 (100.63) | 173.07 (91.86) | -23.51 | (-40.90, -6.13) | 0.009 |  |
| NfL | 6 | 101 | 79.86 (71.08) | 77.72 (51.02) | -2.14 | (-11.65, 7.36) | 0.660 |  |
| P-tau217 | 12 | 68 | 8.89 (2.94) | 5.32 (3.24) | -3.58 | (-4.27, -2.88) | <0.001 |  |
| P-tau181 | 12 | 68 | 7.82 (2.50) | 5.83 (2.98) | -1.99 | (-2.58, -1.41) | <0.001 |  |
| Aβ42 | 12 | 68 | 5.77 (1.85) | 6.93 (1.95) | 1.16 | (0.61, 1.71) | <0.001 |  |
| Aβ40 | 12 | 68 | 134.20 (32.28) | 131.24 (26.89) | -2.96 | (-10.04, 4.12) | 0.416 |  |
| Aβ42/40 | 12 | 68 | 0.04 (0.01) | 0.05 (0.02) | 0.01 | (0.01, 0.01) | <0.001 |  |
| P-tau217/Aβ42 | 12 | 68 | 1.91 (1.95) | 0.90 (0.74) | -1.01 | (-1.48, -0.54) | <0.001 |  |
| P-tau181/Aβ42 | 12 | 68 | 1.70 (1.72) | 0.98 (0.72) | -0.73 | (-1.12, -0.33) | <0.001 |  |
| GFAP | 12 | 68 | 198.09 (104.42) | 174.19 (102.77) | -23.90 | (-52.28, 4.49) | 0.104 |  |
| NfL | 12 | 68 | 70.90 (67.71) | 65.11 (37.07) | -5.79 | (-20.77, 9.20) | 0.452 |  |
| Abbreviations: Aβ, Amyloid-β; CI, confidence interval; GFAP, glial fibrillary acidic protein; NfL, neurofilament light chain; P-tau, phosphorylated tau. | | | | | | | |  |
|  |  |  |  |  |  |  |  |  |

| **Table S5. Regional amyloid PET SUVR alterations at Month 12 versus baseline.** | | | | | | |
| --- | --- | --- | --- | --- | --- | --- |
| **Region** | **N** | **Baseline** | **Month 12** | **Mean change** | **95% CI** | ***P* value** |
| Frontal cortex | 27 | 1.86 (0.36) | 1.39 (0.29) | -0.47 | (-0.60, -0.33) | <0.001 |
| Parietal cortex | 27 | 2.00 (0.38) | 1.49 (0.33) | -0.51 | (-0.66, -0.36) | <0.001 |
| Temporal cortex | 27 | 1.71 (0.31) | 1.29 (0.28) | -0.42 | (-0.54, -0.30) | <0.001 |
| Occipital cortex | 27 | 1.73 (0.32) | 1.37 (0.29) | -0.37 | (-0.50, -0.24) | <0.001 |
| Posterior cingulate | 27 | 1.95 (0.38) | 1.46 (0.32) | -0.49 | (-0.65, -0.33) | <0.001 |
| Global | 27 | 1.74 (0.34) | 1.35 (0.22) | -0.38 | (-0.51, -0.26) | <0.001 |
| Abbreviations: SUVR, standardized uptake value ratio. | | | | | | |

| **Table S6. Standardized response means for clinical and biomarker outcomes at Months 3, 6, and 12.** | | | | | |  |
| --- | --- | --- | --- | --- | --- | --- |
| **Outcome** | **Visit (M)** | **N** | **Mean change** | **SD of change** | **SRM** |  |
| MMSE | 3 | 139 | 0.01 | 1.53 | 0.00 |  |
| MMSE | 6 | 109 | 0.10 | 1.19 | 0.08 |  |
| MMSE | 12 | 68 | 0.57 | 2.02 | 0.28 |  |
| MoCA | 3 | 139 | -0.03 | 1.49 | -0.02 |  |
| MoCA | 6 | 109 | 0.24 | 1.84 | 0.13 |  |
| MoCA | 12 | 68 | 0.35 | 1.96 | 0.18 |  |
| ADAS-Cog13 | 3 | 135 | -0.01 | 1.21 | -0.01 |  |
| ADAS-Cog13 | 6 | 106 | -0.75 | 1.39 | -0.54 |  |
| ADAS-Cog13 | 12 | 68 | -0.94 | 2.21 | -0.43 |  |
| ADCS-ADL | 3 | 135 | 0.13 | 2.03 | 0.07 |  |
| ADCS-ADL | 6 | 106 | 0.86 | 1.56 | 0.55 |  |
| ADCS-ADL | 12 | 68 | 0.85 | 2.53 | 0.34 |  |
| ADCS-iADL | 3 | 135 | 0.23 | 2.07 | 0.11 |  |
| ADCS-iADL | 6 | 107 | 0.65 | 5.35 | 0.12 |  |
| ADCS-iADL | 12 | 68 | 2.59 | 2.62 | 0.99 |  |
| FAQ | 3 | 135 | -0.04 | 0.48 | -0.08 |  |
| FAQ | 6 | 106 | -0.45 | 1.05 | -0.43 |  |
| FAQ | 12 | 68 | -1.21 | 1.72 | -0.70 |  |
| CDR-SB | 3 | 137 | -0.06 | 0.57 | -0.10 |  |
| CDR-SB | 6 | 106 | -0.27 | 0.64 | -0.43 |  |
| CDR-SB | 12 | 68 | -0.47 | 0.76 | -0.62 |  |
| iADRS | 3 | 135 | 0.24 | 3.18 | 0.07 |  |
| iADRS | 6 | 107 | 1.50 | 4.68 | 0.32 |  |
| iADRS | 12 | 68 | 3.49 | 3.57 | 0.98 |  |
| P-tau217 | 3 | 139 | -0.81 | 2.72 | -0.30 |  |
| P-tau217 | 6 | 102 | -2.39 | 2.59 | -0.92 |  |
| P-tau217 | 12 | 68 | -3.57 | 2.91 | -1.23 |  |
| P-tau181 | 3 | 138 | -0.58 | 2.37 | -0.25 |  |
| P-tau181 | 6 | 101 | -1.39 | 2.20 | -0.63 |  |
| P-tau181 | 12 | 68 | -1.99 | 2.45 | -0.81 |  |
| Aβ42 | 3 | 139 | 0.41 | 1.87 | 0.22 |  |
| Aβ42 | 6 | 102 | 0.74 | 2.12 | 0.35 |  |
| Aβ42 | 12 | 68 | 1.16 | 2.33 | 0.50 |  |
| Aβ40 | 3 | 139 | 0.37 | 32.38 | 0.01 |  |
| Aβ40 | 6 | 102 | -6.97 | 36.57 | -0.19 |  |
| Aβ40 | 12 | 68 | -2.96 | 29.80 | -0.10 |  |
| Aβ42/40 | 3 | 139 | 0.00 | 0.01 | 0.22 |  |
| Aβ42/40 | 6 | 102 | 0.01 | 0.02 | 0.53 |  |
| Aβ42/40 | 12 | 68 | 0.01 | 0.02 | 0.60 |  |
| P-tau217/Aβ42 | 3 | 139 | -0.45 | 1.67 | -0.27 |  |
| P-tau217/Aβ42 | 6 | 102 | -0.92 | 1.97 | -0.47 |  |
| P-tau217/Aβ42 | 12 | 68 | -1.01 | 1.98 | -0.51 |  |
| P-tau181/Aβ42 | 3 | 138 | -0.37 | 1.43 | -0.26 |  |
| P-tau181/Aβ42 | 6 | 101 | -0.69 | 1.70 | -0.41 |  |
| P-tau181/Aβ42 | 12 | 68 | -0.72 | 1.68 | -0.43 |  |
| GFAP | 3 | 137 | -23.81 | 82.06 | -0.29 |  |
| GFAP | 6 | 102 | -23.51 | 89.59 | -0.26 |  |
| GFAP | 12 | 68 | -23.90 | 119.44 | -0.20 |  |
| NfL | 3 | 137 | -3.04 | 28.41 | -0.11 |  |
| NfL | 6 | 101 | -2.14 | 48.74 | -0.04 |  |
| NfL | 12 | 68 | -5.79 | 63.05 | -0.09 |  |
| Abbreviations: ADAS-Cog13, 13-item cognitive subscale of the Alzheimer's Disease Assessment Scale; ADCS-ADL, Alzheimer's Disease Cooperative Study—Activities of Daily Living; ADCS-iADL, Alzheimer's Disease Cooperative Study—Instrumental Activities of Daily Living; Aβ, Amyloid-β; CDR-SB, sum of boxes of the Clinical Dementia Rating Scale; FAQ, Functional Activities Questionnaire; GFAP, glial fibrillary acidic protein; iADRS, Integrated Alzheimer's Disease Rating Scale; MMSE, Mini-Mental State Examination; MoCA, The Montreal Cognitive Assessment; NfL, neurofilament light chain; P-tau, phosphorylated tau. | | | | | |  |
|  |  |  |  |  |  |  |
|  |  |  |  |  |  |  |
|  |  |  |  |  |  |  |
|  |  |  |  |  |  |  |
|  |  |  |  |  |  |  |

| **Table S7. Subgroup analyses of key outcomes at Month 3, Month 6, and Month 12.** | | | | | | | | |  |
| --- | --- | --- | --- | --- | --- | --- | --- | --- | --- |
| **Subgroup factor** | **Subgroup** | **Outcome** | **Visit (M)** | **N** | **Mean change** | **SD** | **Within-group *P* value** | **Interaction *P* value** |  |
| Age | ≤74 years | ADAS-Cog13 | 3 | 96 | -0.03 | 1.39 | 0.826 | 0.720 |  |
| Age | ≥75 years | ADAS-Cog13 | 3 | 39 | 0.05 | 0.56 | 0.570 |  |  |
| Age | ≤74 years | ADAS-Cog13 | 6 | 73 | -0.82 | 1.49 | <0.001 | 0.400 |  |
| Age | ≥75 years | ADAS-Cog13 | 6 | 33 | -0.58 | 1.15 | 0.007 |  |  |
| Age | ≤74 years | ADAS-Cog13 | 12 | 48 | -0.83 | 2.35 | 0.018 | 0.537 |  |
| Age | ≥75 years | ADAS-Cog13 | 12 | 20 | -1.20 | 1.88 | 0.010 |  |  |
| Age | ≤74 years | iADRS | 3 | 96 | 0.34 | 3.75 | 0.371 | 0.543 |  |
| Age | ≥75 years | iADRS | 3 | 39 | -0.03 | 0.67 | 0.812 |  |  |
| Age | ≤74 years | iADRS | 6 | 74 | 1.27 | 5.38 | 0.046 | 0.440 |  |
| Age | ≥75 years | iADRS | 6 | 33 | 2.03 | 2.43 | <0.001 |  |  |
| Age | ≤74 years | iADRS | 12 | 48 | 3.56 | 3.77 | <0.001 | 0.785 |  |
| Age | ≥75 years | iADRS | 12 | 20 | 3.30 | 3.13 | <0.001 |  |  |
| Age | ≤74 years | CDR-SB | 3 | 97 | -0.10 | 0.64 | 0.114 | 0.151 |  |
| Age | ≥75 years | CDR-SB | 3 | 40 | 0.05 | 0.32 | 0.323 |  |  |
| Age | ≤74 years | CDR-SB | 6 | 73 | -0.22 | 0.66 | 0.006 | 0.195 |  |
| Age | ≥75 years | CDR-SB | 6 | 33 | -0.39 | 0.58 | <0.001 |  |  |
| Age | ≤74 years | CDR-SB | 12 | 48 | -0.43 | 0.84 | <0.001 | 0.470 |  |
| Age | ≥75 years | CDR-SB | 12 | 20 | -0.58 | 0.55 | <0.001 |  |  |
| Age | ≤74 years | P-tau217 | 3 | 98 | -0.77 | 2.17 | <0.001 | 0.786 |  |
| Age | ≥75 years | P-tau217 | 3 | 41 | -0.91 | 3.75 | 0.128 |  |  |
| Age | ≤74 years | P-tau217 | 6 | 69 | -2.24 | 2.29 | <0.001 | 0.407 |  |
| Age | ≥75 years | P-tau217 | 6 | 33 | -2.70 | 3.13 | <0.001 |  |  |
| Age | ≤74 years | P-tau217 | 12 | 48 | -3.37 | 2.73 | <0.001 | 0.360 |  |
| Age | ≥75 years | P-tau217 | 12 | 20 | -4.08 | 3.32 | <0.001 |  |  |
| Age | ≤74 years | Aβ42/40 | 3 | 98 | 0.00 | 0.01 | 0.080 | 0.296 |  |
| Age | ≥75 years | Aβ42/40 | 3 | 41 | 0.00 | 0.02 | 0.064 |  |  |
| Age | ≤74 years | Aβ42/40 | 6 | 69 | 0.01 | 0.01 | <0.001 | 0.571 |  |
| Age | ≥75 years | Aβ42/40 | 6 | 33 | 0.01 | 0.02 | 0.054 |  |  |
| Age | ≤74 years | Aβ42/40 | 12 | 48 | 0.01 | 0.02 | <0.001 | 0.137 |  |
| Age | ≥75 years | Aβ42/40 | 12 | 20 | 0.01 | 0.02 | 0.134 |  |  |
| Sex | Female | ADAS-Cog13 | 3 | 82 | -0.05 | 1.52 | 0.772 | 0.622 |  |
| Sex | Male | ADAS-Cog13 | 3 | 53 | 0.06 | 0.36 | 0.261 |  |  |
| Sex | Female | ADAS-Cog13 | 6 | 67 | -0.76 | 1.38 | <0.001 | 0.878 |  |
| Sex | Male | ADAS-Cog13 | 6 | 39 | -0.72 | 1.41 | 0.003 |  |  |
| Sex | Female | ADAS-Cog13 | 12 | 42 | -0.98 | 2.49 | 0.015 | 0.870 |  |
| Sex | Male | ADAS-Cog13 | 12 | 26 | -0.89 | 1.71 | 0.014 |  |  |
| Sex | Female | iADRS | 3 | 82 | 0.40 | 4.07 | 0.373 | 0.455 |  |
| Sex | Male | iADRS | 3 | 53 | -0.02 | 0.42 | 0.742 |  |  |
| Sex | Female | iADRS | 6 | 68 | 1.00 | 5.38 | 0.130 | 0.141 |  |
| Sex | Male | iADRS | 6 | 39 | 2.39 | 2.95 | <0.001 |  |  |
| Sex | Female | iADRS | 12 | 42 | 4.02 | 4.00 | <0.001 | 0.115 |  |
| Sex | Male | iADRS | 12 | 26 | 2.62 | 2.59 | <0.001 |  |  |
| Sex | Female | CDR-SB | 3 | 83 | -0.05 | 0.70 | 0.533 | 0.795 |  |
| Sex | Male | CDR-SB | 3 | 54 | -0.07 | 0.25 | 0.031 |  |  |
| Sex | Female | CDR-SB | 6 | 67 | -0.27 | 0.70 | 0.003 | 0.918 |  |
| Sex | Male | CDR-SB | 6 | 39 | -0.28 | 0.52 | 0.002 |  |  |
| Sex | Female | CDR-SB | 12 | 42 | -0.43 | 0.79 | 0.001 | 0.567 |  |
| Sex | Male | CDR-SB | 12 | 26 | -0.54 | 0.72 | <0.001 |  |  |
| Sex | Female | P-tau217 | 3 | 83 | -0.96 | 2.25 | <0.001 | 0.443 |  |
| Sex | Male | P-tau217 | 3 | 56 | -0.60 | 3.30 | 0.182 |  |  |
| Sex | Female | P-tau217 | 6 | 65 | -2.72 | 2.66 | <0.001 | 0.089 |  |
| Sex | Male | P-tau217 | 6 | 37 | -1.81 | 2.39 | <0.001 |  |  |
| Sex | Female | P-tau217 | 12 | 42 | -3.91 | 3.23 | <0.001 | 0.237 |  |
| Sex | Male | P-tau217 | 12 | 26 | -3.04 | 2.27 | <0.001 |  |  |
| Sex | Female | Aβ42/40 | 3 | 83 | 0.00 | 0.01 | 0.047 | 0.681 |  |
| Sex | Male | Aβ42/40 | 3 | 56 | 0.00 | 0.01 | 0.105 |  |  |
| Sex | Female | Aβ42/40 | 6 | 65 | 0.01 | 0.02 | <0.001 | 0.147 |  |
| Sex | Male | Aβ42/40 | 6 | 37 | 0.01 | 0.02 | 0.068 |  |  |
| Sex | Female | Aβ42/40 | 12 | 42 | 0.01 | 0.02 | <0.001 | 0.828 |  |
| Sex | Male | Aβ42/40 | 12 | 26 | 0.01 | 0.02 | 0.004 |  |  |
| APOE ε4 status | Carrier | ADAS-Cog13 | 3 | 74 | -0.11 | 1.58 | 0.557 | 0.287 |  |
| APOE ε4 status | Non-carrier | ADAS-Cog13 | 3 | 61 | 0.11 | 0.45 | 0.051 |  |  |
| APOE ε4 status | Carrier | ADAS-Cog13 | 6 | 56 | -0.66 | 1.25 | <0.001 | 0.509 |  |
| APOE ε4 status | Non-carrier | ADAS-Cog13 | 6 | 50 | -0.84 | 1.53 | <0.001 |  |  |
| APOE ε4 status | Carrier | ADAS-Cog13 | 12 | 33 | -0.64 | 1.73 | 0.042 | 0.273 |  |
| APOE ε4 status | Non-carrier | ADAS-Cog13 | 12 | 35 | -1.23 | 2.58 | 0.008 |  |  |
| APOE ε4 status | Carrier | iADRS | 3 | 74 | 0.45 | 4.23 | 0.367 | 0.403 |  |
| APOE ε4 status | Non-carrier | iADRS | 3 | 61 | -0.02 | 0.85 | 0.880 |  |  |
| APOE ε4 status | Carrier | iADRS | 6 | 57 | 0.61 | 5.54 | 0.406 | 0.035 |  |
| APOE ε4 status | Non-carrier | iADRS | 6 | 50 | 2.52 | 3.20 | <0.001 |  |  |
| APOE ε4 status | Carrier | iADRS | 12 | 33 | 2.24 | 2.83 | <0.001 | 0.005 |  |
| APOE ε4 status | Non-carrier | iADRS | 12 | 35 | 4.66 | 3.83 | <0.001 |  |  |
| APOE ε4 status | Carrier | CDR-SB | 3 | 75 | -0.03 | 0.65 | 0.660 | 0.570 |  |
| APOE ε4 status | Non-carrier | CDR-SB | 3 | 62 | -0.09 | 0.44 | 0.117 |  |  |
| APOE ε4 status | Carrier | CDR-SB | 6 | 56 | -0.07 | 0.56 | 0.344 | <0.001 |  |
| APOE ε4 status | Non-carrier | CDR-SB | 6 | 50 | -0.50 | 0.66 | <0.001 |  |  |
| APOE ε4 status | Carrier | CDR-SB | 12 | 33 | -0.26 | 0.78 | 0.067 | 0.024 |  |
| APOE ε4 status | Non-carrier | CDR-SB | 12 | 35 | -0.67 | 0.70 | <0.001 |  |  |
| APOE ε4 status | Carrier | P-tau217 | 3 | 77 | -0.89 | 2.26 | <0.001 | 0.708 |  |
| APOE ε4 status | Non-carrier | P-tau217 | 3 | 62 | -0.72 | 3.21 | 0.084 |  |  |
| APOE ε4 status | Carrier | P-tau217 | 6 | 53 | -2.42 | 2.80 | <0.001 | 0.927 |  |
| APOE ε4 status | Non-carrier | P-tau217 | 6 | 49 | -2.37 | 2.36 | <0.001 |  |  |
| APOE ε4 status | Carrier | P-tau217 | 12 | 33 | -3.52 | 2.99 | <0.001 | 0.885 |  |
| APOE ε4 status | Non-carrier | P-tau217 | 12 | 35 | -3.63 | 2.88 | <0.001 |  |  |
| APOE ε4 status | Carrier | Aβ42/40 | 3 | 77 | 0.00 | 0.01 | 0.097 | 0.840 |  |
| APOE ε4 status | Non-carrier | Aβ42/40 | 3 | 62 | 0.00 | 0.01 | 0.046 |  |  |
| APOE ε4 status | Carrier | Aβ42/40 | 6 | 53 | 0.01 | 0.02 | <0.001 | 0.646 |  |
| APOE ε4 status | Non-carrier | Aβ42/40 | 6 | 49 | 0.01 | 0.02 | 0.002 |  |  |
| APOE ε4 status | Carrier | Aβ42/40 | 12 | 33 | 0.01 | 0.02 | <0.001 | 0.987 |  |
| APOE ε4 status | Non-carrier | Aβ42/40 | 12 | 35 | 0.01 | 0.02 | 0.003 |  |  |
| Disease stage | MCI/Mild dementia | ADAS-Cog13 | 3 | 99 | 0.12 | 0.48 | 0.014 | 0.039 |  |
| Disease stage | Moderate-to-severe dementia | ADAS-Cog13 | 3 | 36 | -0.36 | 2.18 | 0.327 |  |  |
| Disease stage | MCI/Mild dementia | ADAS-Cog13 | 6 | 79 | -0.90 | 1.48 | <0.001 | 0.051 |  |
| Disease stage | Moderate-to-severe dementia | ADAS-Cog13 | 6 | 27 | -0.30 | 0.95 | 0.118 |  |  |
| Disease stage | MCI/Mild dementia | ADAS-Cog13 | 12 | 52 | -1.31 | 2.26 | <0.001 | 0.013 |  |
| Disease stage | Moderate-to-severe dementia | ADAS-Cog13 | 12 | 16 | 0.25 | 1.57 | 0.534 |  |  |
| Disease stage | MCI/Mild dementia | iADRS | 3 | 99 | -0.05 | 0.80 | 0.531 | 0.082 |  |
| Disease stage | Moderate-to-severe dementia | iADRS | 3 | 36 | 1.03 | 6.01 | 0.312 |  |  |
| Disease stage | MCI/Mild dementia | iADRS | 6 | 80 | 1.93 | 5.26 | 0.002 | 0.110 |  |
| Disease stage | Moderate-to-severe dementia | iADRS | 6 | 27 | 0.26 | 1.70 | 0.435 |  |  |
| Disease stage | MCI/Mild dementia | iADRS | 12 | 52 | 3.65 | 3.68 | <0.001 | 0.487 |  |
| Disease stage | Moderate-to-severe dementia | iADRS | 12 | 16 | 2.94 | 3.26 | 0.003 |  |  |
| Disease stage | MCI/Mild dementia | CDR-SB | 3 | 99 | -0.01 | 0.42 | 0.810 | 0.107 |  |
| Disease stage | Moderate-to-severe dementia | CDR-SB | 3 | 38 | -0.18 | 0.83 | 0.181 |  |  |
| Disease stage | MCI/Mild dementia | CDR-SB | 6 | 79 | -0.25 | 0.60 | <0.001 | 0.577 |  |
| Disease stage | Moderate-to-severe dementia | CDR-SB | 6 | 27 | -0.33 | 0.75 | 0.028 |  |  |
| Disease stage | MCI/Mild dementia | CDR-SB | 12 | 52 | -0.58 | 0.71 | <0.001 | 0.037 |  |
| Disease stage | Moderate-to-severe dementia | CDR-SB | 12 | 16 | -0.13 | 0.85 | 0.564 |  |  |
| Disease stage | MCI/Mild dementia | P-tau217 | 3 | 101 | -0.95 | 2.67 | <0.001 | 0.330 |  |
| Disease stage | Moderate-to-severe dementia | P-tau217 | 3 | 38 | -0.45 | 2.83 | 0.339 |  |  |
| Disease stage | MCI/Mild dementia | P-tau217 | 6 | 74 | -2.58 | 2.64 | <0.001 | 0.227 |  |
| Disease stage | Moderate-to-severe dementia | P-tau217 | 6 | 28 | -1.89 | 2.43 | <0.001 |  |  |
| Disease stage | MCI/Mild dementia | P-tau217 | 12 | 52 | -4.14 | 2.69 | <0.001 | 0.003 |  |
| Disease stage | Moderate-to-severe dementia | P-tau217 | 12 | 16 | -1.73 | 2.92 | 0.032 |  |  |
| Disease stage | MCI/Mild dementia | Aβ42/40 | 3 | 101 | 0.00 | 0.01 | 0.242 | 0.065 |  |
| Disease stage | Moderate-to-severe dementia | Aβ42/40 | 3 | 38 | 0.01 | 0.01 | <0.001 |  |  |
| Disease stage | MCI/Mild dementia | Aβ42/40 | 6 | 74 | 0.01 | 0.02 | <0.001 | 0.238 |  |
| Disease stage | Moderate-to-severe dementia | Aβ42/40 | 6 | 28 | 0.01 | 0.01 | <0.001 |  |  |
| Disease stage | MCI/Mild dementia | Aβ42/40 | 12 | 52 | 0.01 | 0.02 | <0.001 | 0.830 |  |
| Disease stage | Moderate-to-severe dementia | Aβ42/40 | 12 | 16 | 0.01 | 0.02 | 0.030 |  |  |
| Abbreviations: ADAS-Cog13, 13-item cognitive subscale of the Alzheimer's Disease Assessment Scale; APOE, apolipoprotein E; Aβ, Amyloid-β; CDR-SB, sum of boxes of the Clinical Dementia Rating Scale; iADRS, Integrated Alzheimer's Disease Rating Scale; MCI, mild cognitive impairment; P-tau, phosphorylated tau. | | | | | | | | |  |
|  |  |  |  |  |  |  |  |  |  |

**Table S8. Safety and ARIA in the overall cohort, follow-up strata, documented dropouts, and moderate-to-severe dementia.**

| **Group** | **Event** | **n/N (%)** | **95% CI** |
| --- | --- | --- | --- |
| Overall cohort | Any infusion-related reaction | 12/181 (6.6) | 3.83-11.23 |
| Overall cohort | Any ARIA | 13/181 (7.2) | 4.25-11.90 |
| Overall cohort | ARIA-H | 10/181 (5.5) | 3.03-9.87 |
| Overall cohort | ARIA-E with or without H | 3/181 (1.7) | 0.57-4.76 |
| Month 12 completers | Any infusion-related reaction | 3/68 (4.4) | 1.51-12.19 |
| Month 12 completers | Any ARIA | 3/68 (4.4) | 1.51-12.19 |
| Month 12 completers | ARIA-H | 3/68 (4.4) | 1.51-12.19 |
| Month 12 completers | ARIA-E with or without H | 0/68 (0.0) | 0.00-5.35 |
| No Month 12 data | Any infusion-related reaction | 9/113 (8.0) | 4.25-14.45 |
| No Month 12 data | Any ARIA | 10/113 (8.9) | 4.88-15.53 |
| No Month 12 data | ARIA-H | 7/113 (6.2) | 3.03-12.24 |
| No Month 12 data | ARIA-E with or without H | 3/113 (2.7) | 0.91-7.52 |
| Documented dropouts | Any infusion-related reaction | 4/37 (10.8) | 4.29-24.71 |
| Documented dropouts | Any ARIA | 10/37 (27.0) | 15.40-42.98 |
| Documented dropouts | ARIA-H | 7/37 (18.9) | 9.48-34.20 |
| Documented dropouts | ARIA-E with or without H | 3/37 (8.1) | 2.80-21.30 |
| No documented dropout | Any infusion-related reaction | 8/144 (5.6) | 2.84-10.58 |
| No documented dropout | Any ARIA | 3/144 (2.1) | 0.71-5.95 |
| No documented dropout | ARIA-H | 3/144 (2.1) | 0.71-5.95 |
| No documented dropout | ARIA-E with or without H | 0/144 (0.0) | 0.00-2.60 |
| Moderate-to-severe AD dementia | Any infusion-related reaction | 4/45 (8.9) | 3.51-20.73 |
| Moderate-to-severe AD dementia | Any ARIA | 3/45 (6.7) | 2.29-17.86 |
| Moderate-to-severe AD dementia | ARIA-H | 3/45 (6.7) | 2.29-17.86 |
| Moderate-to-severe AD dementia | ARIA-E with or without H | 0/45 (0.0) | 0.00-7.87 |
| Overall cohort | Other medical conditions | 11/181 (6.1) | 3.43-10.55 |
| Overall cohort | ARIA-related discontinuation | 10/181 (5.5) | 3.03-9.87 |
| Overall cohort | Transfer to another hospital | 8/181 (4.4) | 2.26-8.48 |
| Overall cohort | Unable to continue paying for lecanemab | 6/181 (3.3) | 1.53-7.04 |
| Overall cohort | Death | 2/181 (1.1) | 0.30-3.94 |

ARIA-E includes ARIA-E with or without concomitant ARIA-H. ARIA-H denotes ARIA-H without ARIA-E.

Supplementary figure legends

Figure S1. Continuous clinical outcome changes from baseline. Points show mean changes and bars show 95% confidence intervals.

Figure S2. Continuous plasma biomarker changes from baseline. Points show mean changes and bars show 95% confidence intervals.

Figure S3. Paired global amyloid PET SUVR from baseline to Month 12. Thin lines show individual participants and the thick line shows the mean with 95% confidence intervals.

Figure S4. Exploratory continuous treatment signals in moderate-to-severe AD dementia. Mean changes in selected outcomes are oriented so that positive values indicate improvement; Aβ42/40 changes are multiplied by 100 for display. Points show means and bars show 95% confidence intervals.

Figure S5. Safety and documented dropout. (A) ARIA and infusion-related reactions by follow-up and dropout strata. (B) Documented dropout reasons.
